# Multi-omic definition of metabolic obesity through adipose tissue–microbiome interactions

**DOI:** 10.1038/s41591-025-04009-7

**Published:** 2026-01-02

**Authors:** Rima M. Chakaroun, Meenakshi Pradhan, Elias Björnson, Daniel Arvidsson, Jonatan Fridolfsson, Anders Gummesson, Marc Schoeler, Matthias Mitteregger, Gustav J. Smith, Ingrid Larsson, Mats Börjesson, Matthias Blüher, Mathias Uhlén, Michael Stumvoll, Göran Bergström, Valentina Tremaroli, Fredrik Bäckhed

**Affiliations:** 1 Wallenberg Laboratory, Department of Molecular and Clinical Medicine, Institute of Medicine, University of Gothenburg, Gothenburg, Sweden; 2 Medical Department III – Endocrinology, Nephrology, Rheumatology, University of Leipzig Medical Center, Leipzig, Germany; 3 Department of Clinical Physiology Region Västra Götaland, Sahlgrenska University Hospital, Gothenburg, Sweden; 4 Center for Health and Performance, Department of Food and Nutrition and Sport Science, University of Gothenburg, Gothenburg, Sweden; 5 Center for Lifestyle Intervention, Department of Molecular and Clinical Medicine, Institute of Medicine, Sahlgrenska Academy, University of Gothenburg, Gothenburg, Sweden; 6 Sahlgrenska University Hospital, Region Västra Götaland, Gothenburg, Sweden; 7 Department of Clinical Genetics and Genomics, Sahlgrenska University Hospital, Gothenburg, Sweden; 8 Department of Cardiology, Sahlgrenska University Hospital, Gothenburg, Sweden; 9 Department of Cardiology, Clinical Sciences, Lund University and Skåne University Hospital, Lund, Sweden; 10 Wallenberg Center for Molecular Medicine and Lund University Diabetes Center, Lund University, Lund, Sweden; 11 Department of Medicine, Sahlgrenska University Hospital, Gothenburg, Sweden; 12 Institute of Internal Medicine and Clinical Nutrition, Sahlgrenska Academy, University of Gothenburg, Gothenburg, Sweden; 13 Department of MGAÖ, Sahlgrenska University Hospital, Region of Västra Götaland, Gothenburg, Sweden; 14 Helmholtz Institute for Metabolic, Obesity and Vascular Research (HI-MAG) of the Helmholtz Zentrum München at the University of Leipzig and University Hospital Leipzig, Leipzig, Germany; 15 LeiCeM − Leipzig Center of Metabolism, Leipzig University, Leipzig, Germany; 16 Science for Life Laboratory, Department of Protein Science, KTH-Royal Institute of Technology, Stockholm, Sweden; 17 Novo Nordisk Foundation Microbiome Health Initiative and the National Food Institute, Technical University of Denmark, Kongens Lyngby, Denmark

**Keywords:** Endocrine system and metabolic diseases, Metabolic disorders, Microbiology, Bioinformatics

## Abstract

Obesity’s metabolic heterogeneity is not fully captured by body mass index (BMI). Here we show that deep multi-omics phenotyping of 1,408 individuals defines a metabolome-informed obesity metric (metBMI) that captures adipose tissue-related dysfunction across organ systems. In an external cohort (*n* = 466), metBMI explained 52% of BMI variance and more accurately reflected adiposity than other omics models. Individuals with higher-than-expected metBMI had 2–5-fold higher odds of fatty liver disease, diabetes, severe visceral fat accumulation and attenuation, insulin resistance, hyperinsulinemia and inflammation and, in bariatric surgery (*n* = 75), achieved 30% less weight loss. This obesogenic signature aligned with reduced microbiome richness, altered ecology and functional potential. A 66-metabolite panel retained 38.6% explanatory power, with 90% covarying with the microbiome. Mediation analysis revealed a bidirectional, metabolite-centered host–microbiome axis, mediated by lipids, amino acids and diet-derived metabolites. These findings define an adipose-linked, microbiome-connected metabolic signature that outperforms BMI in stratifying cardiometabolic risk and guiding precision interventions.

## Abstract (web summary)

Metabolomics, metagenomics, proteins and genetics were combined with clinical data to define a metabolic signature of obesity.

## Main

Obesity is increasingly recognized as a chronic, multifactorial and progressive disease, driven by excess adiposity and leading to dysfunction at the tissue, organ and whole-body levels^1,2^. It is the leading cause of type 2 diabetes (T2D) and a significant contributor to cardiometabolic morbidity and mortality^3^. However, diagnosis still relies on BMI—a surrogate with limited capacity to capture individual cardiometabolic risk^4^. Indeed, 20–30% of individuals with T2D do not suffer from BMI-defined obesity^5^, and a significant number of global cardiovascular deaths linked to abnormal BMI occur in those below the obesity threshold^6^. This has prompted calls to refine diagnostic criteria to prevent undertreatment of at-risk individuals not identified by BMI^2,7^.

Although BMI may miss functional changes associated with obesity, multi-omics approaches offer a metabolically informed view of health by integrating signals across organs and systems, enabling more precise characterization of obesity-related risk and clinically meaningful obesity heterogeneity^8,9^. Circulating metabolites, shaped by host genetics, diet and the gut microbiome, offer a systems-level readout of metabolic health beyond excess weight^8,10^: an obesogenic metabolite signature is linked to a two-fold higher risk of future T2D, up to a five-fold increase in cardiovascular events and an 80% increase in mortality^9^, highlighting the potential of metabolomics for early risk stratification^8,9^. However, the phenotypic diversity underlying this signature and its drivers remains insufficiently defined.

The gut microbiome is interlinked with host metabolism and contributes to approximately 15% of circulating metabolite levels in healthy individuals^11,12^, rising nearly to 30% in prediabetes and T2D^13^, with several microbiota-derived metabolites causally implicated in cardiometabolic risk^14^. Conversely, up to 60% of the variation in gut microbiome diversity is explained by the circulating metabolome^15^, underscoring bidirectional host–microbial metabolic interplay. In obesity and related metabolic disorders, bacterial diversity is reduced and functional capacity altered^16–18^. Accordingly, the circulating metabolome may serve as a proxy for microbiome-derived signals, with disrupted interactions contributing to the metabolic heterogeneity across the BMI spectrum.

**Extended Data Fig. 1:**
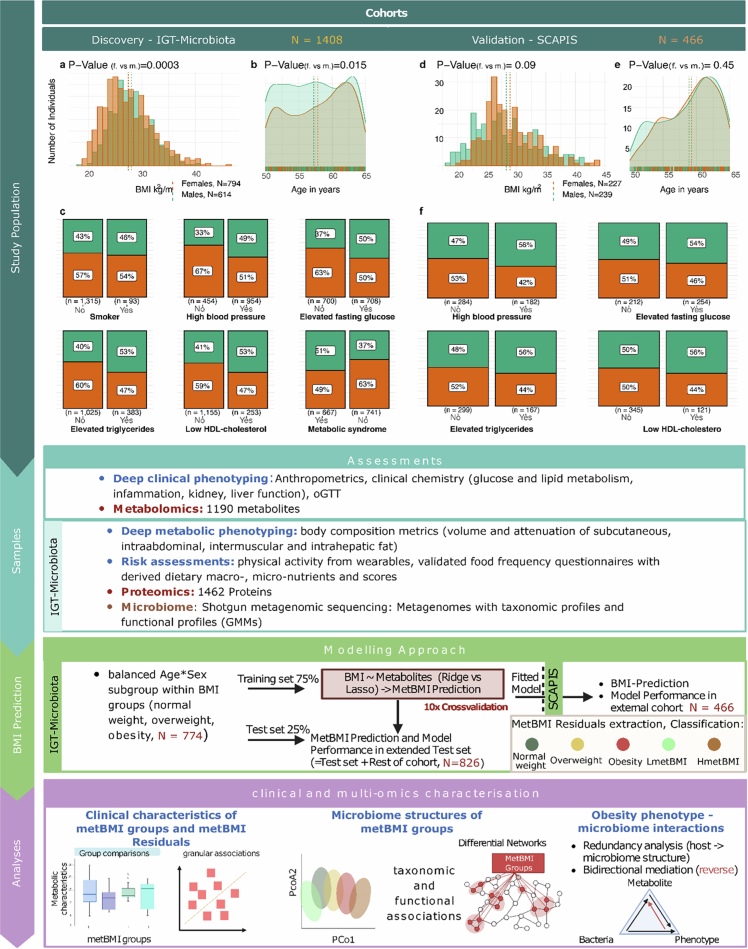
Study overview. Distribution of BMI (**a,d**), age (**b,e**), and metabolic features (**c,f**). N, number of participants. Solid and dashed lines indicate kernel density estimates, BMI and age means. *P* values of two-sided Wilcoxon-rank sum test shown for comparisons between females and males. Summary values available in Supplementary Tables 1 and 3.

Here we hypothesize that a metabolome-informed BMI prediction provides a more precise and biologically grounded measure of adiposity-related risk than traditional BMI. Using machine learning and deep phenotyping from two Swedish cohorts (*n* = 1,408 and *n* = 466; Extended Data Fig. 1), we integrate computed tomography-based adipose tissue quantification and metabolomic, proteomic, genomic and metagenomic data with comprehensive clinical, lifestyle, dietary and physical activity measures. We demonstrate that metBMI captures metabolic dysfunction across the BMI spectrum, predicts bariatric surgery response in an independent cohort (*n* = 75) and reveals potentially causal microbiome–metabolome interactions linked to cardiometabolic risk. This integrative framework advances precision phenotyping of obesity, illuminates inter-organ and inter-organismal disease pathways and may enable earlier, more targeted interventions beyond BMI-defined thresholds.

## Results

### Multi-omics-based modeling of obesity

We first sought to determine which molecular domains—circulating metabolome and proteome, gut metagenome and dietary intake—were most strongly associated with obesity (operationally defined as excess weight relative to height, as still widely applied) and adiposity (reflecting adipose tissue quantity and distribution) in a well-characterized, cross-sectional cohort (Impaired Glucose Tolerance and Microbiota Study (IGT-microbiota); *n* = 1,408; Methods, Supplementary Table 1 and Extended Data Fig. 1). This cohort, comprising at-risk individuals without established cardiovascular disease or diagnosed T2D, enables the delineation of preclinical obesity-related signatures that may generalize to populations with more advanced disease.

Using nested ridge regression with 10-fold cross-validation to optimize model regularization, we trained predictive models for BMI, waist-to-hip ratio (WHR), waist circumference and computed tomography-derived visceral and subcutaneous adipose tissue (VAT and SAT) areas. Models were constructed using individual omics layers—circulating metabolites (*n* = 1,190); proteins (*n* = 1,462); microbiome features such as gut bacterial species (metagenome-assembled genomes (MAGs) (*n* = 2,820)); gut microbial modules (GMMs) (*n* = 117); Kyoto Encyclopedia of Genes and Genomes (KEGG) orthologues (*n* = 11,411, corresponding to 384 pathways); and dietary variables (including dietary indices, macro-nutrient and micro-nutrient intake and food groups)—and further integrated into a combined multi-omics model (*n* = 5,420 variables, including metabolome, proteome, metagenome and diet).

**Fig. 1:**
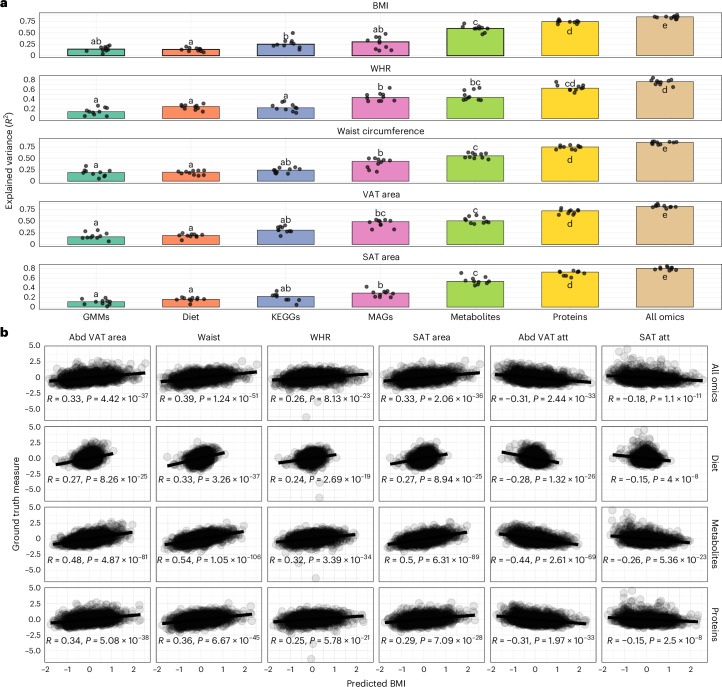
Multi-omics prediction of adiposity. **a**, Proportion of variance explained (hold-out *R*^2^) for traits predicted from single omics layers: GMMs, diet, KEGG orthologues, MAGs, plasma metabolites and proteins or their combination within the IGT-microbiota cohort. Points show the per-fold *R*^2^, and bars summarize the median across ridge regression cross-validation folds (*n* = 10). Letters denote pairwise differences, with bars sharing a letter not differing significantly (two-sided Wilcoxon rank-sum test, Benjamini–Hochberg corrected). Exact *P* values are in Supplementary Table 2. **b**, Two-sided Pearson’s correlations between omics-predicted BMI and ground truth adiposity traits. The line represents the linear regression fit, and each point represents 1 individual with a total *n* = 1,408. Pearson’s *r* correlation coefficient and the corresponding nominal *P* value are shown in each panel. Abd VAT, abdominal visceral adipose tissue; Abd VAT att, abdominal visceral adipose tissue and attenuation; att, attenuation.

**Extended Data Fig. 2:**
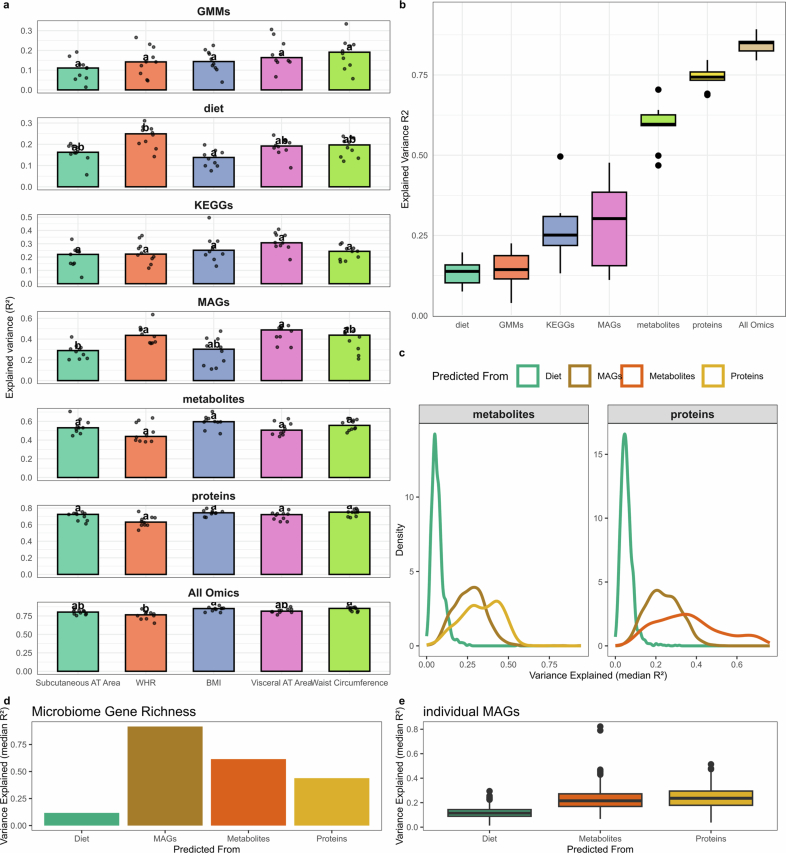
Variance explained (R2) across measures of obesity and adiposity and across omics datasets (inter-omics). **a**. Bar plots show the proportion of variance explained (hold-out R^2^) for each obesity and body-composition trait when predicted from individual data layers: gut-metabolic modules (GMMs), diet, KEGG orthologues, metagenome-assembled genomes (MAGs), serum metabolites, circulating proteins, or their combination (All omics). Each dot is the R² obtained in one-fold of ten-fold ridge-regression cross-validation; bar height is the median across folds (n = 10). Letters indicate pairwise differences: bars that share a letter do not differ significantly (two-sided Wilcoxon rank-sum test, Benjamini–Hochberg adjusted across all feature-space comparisons). *P* values and summary statistics are found under Supplementary Table 2. **b**. Boxplots of variances explained by diet, proteins, metabolites, GMMs, KEGGs, MAGs and all omics combined for BMI. Box plots display the median, interquartile range (IQR) with whiskers specifying ±1.5*IQR of R² distribution per feature obtained in the 10-fold cross-validation (n = 10) and plotted points denoting outliers. Source data under Supplementary Table 2. **c**. Density plots of variance explained (median R²) by predictors across omics layers. Each line represents the distribution of variances explained for features in either the metabolites or proteins feature space, stratified by the predictor used. Colors correspond to the source omics layer used for prediction. **d**. Bar plots show the median proportion of variance explained for microbiome gene richness derived from a 10-fold cross-validation (n = 10), when predicted from individual data layers: diet, metagenome-assembled genomes (MAGs), serum metabolites, and circulating proteins **e**. Boxplots of variance distribution for single metagenomes (MAGs), which could be robustly explained by diet, metabolites, and proteins (1438 MAGs from Diet, 1440 for metabolites and 1440 from proteins). Three R2 values are derived for each MAG in a regression model with 10-fold internal cross- validation and three repeats (n_Diet_= 4318, n_Metabolomics_ = 4320, n_Proteomics_ = 4320). Boxplots show the median and interquartile ranges (IQR); whiskers extend to ±1.5*IQR from the quartiles and plotted points denote outliers. Source Data under Supplementary Table 3.

MAGs explained a similar proportion of variance in central adiposity traits (44% for waist circumference and approximately 50% for VAT area, Bonferroni-adjusted *P* = 1, Wilcoxon rank-sum test against metabolite-based estimates; Fig. 1a, Supplementary Table 2 and Extended Data Fig. 2a), suggesting shared links with visceral fat. However, for BMI, metabolites explained nearly twice the variance captured by MAGs (60% versus 30%, respectively; Fig. 1a and Extended Data Fig. 2b), indicating that the metabolome better represents broader obesity-related processes.

Consistently, the circulating metabolome provided the most physiologically informative signal for predicting obesity among individual omics layers, particularly in capturing the strongest associations with adiposity-related traits (Fig. 1a,b): metabolite-predicted BMI showed significantly stronger correlations with ground truth measures, such as waist circumference and VAT and SAT area, than BMI estimates derived from the proteome, diet or even the combined multi-omics model (Fig. 1b). These results position the metabolome as a more biologically grounded proxy of obesity-related fat accumulation.

The combined multi-omics model achieved the highest overall predictive performance (median variance explained (VE_med_) 0.8 for SAT area to 0.85 for BMI; Fig. 1a and Supplementary Table 2). However, contributions across layers were not additive, reflecting overlapping molecular signals. The second-highest overall predictive performance was observed for the proteome, which explained substantial variance for several traits (for example, VE_med_ 0.74 for BMI and waist and 0.71–0.74 for VAT and SAT; Fig. 1a and Supplementary Table 2). Nonetheless, its performance did not significantly exceed that of the metabolome for several traits (for example, SAT area; Bonferroni-adjusted *P* = 0.3), and the associations with central adiposity traits were less pronounced (Fig. 1b). This observation is further supported by recent intervention data, where proteome-predicted BMI remained stable despite reductions in BMI, metabolite-predicted BMI and improvements in metabolic health, suggesting proteome stability at the expense of metabolic responsiveness to intervention^10^.

Finally, inter-omic comparisons highlighted the broader integrative capacity of the metabolome: metabolites explained up to 76% of the variance of individual proteins (median 35%). In comparison, proteins explained up to 74% of individual metabolites with a similar median of 34% (Extended Data Fig. 2c and Supplementary Table 3). Microbiome gene richness was best explained by metabolites, with a median variance of 61%, compared to 44% for proteins (Extended Data Fig. 2d). Similarly, metabolites outperformed proteins in explaining individual species abundances, reaching a maximum of 82% variance explained for specific MAGs versus a maximum of 51% for proteins (Extended Data Fig. 2e). However, the VE_med_ for MAGs was similar for both metabolites and proteins (22% and 24%, respectively; Extended Data Fig. 2e).

These results underscore strong covariance across omics layers and highlight the metabolome’s central role as a clinically relevant integrator of host, microbial and dietary signals.

### Uncoupling the obesogenic signature from BMI

**Fig. 2:**
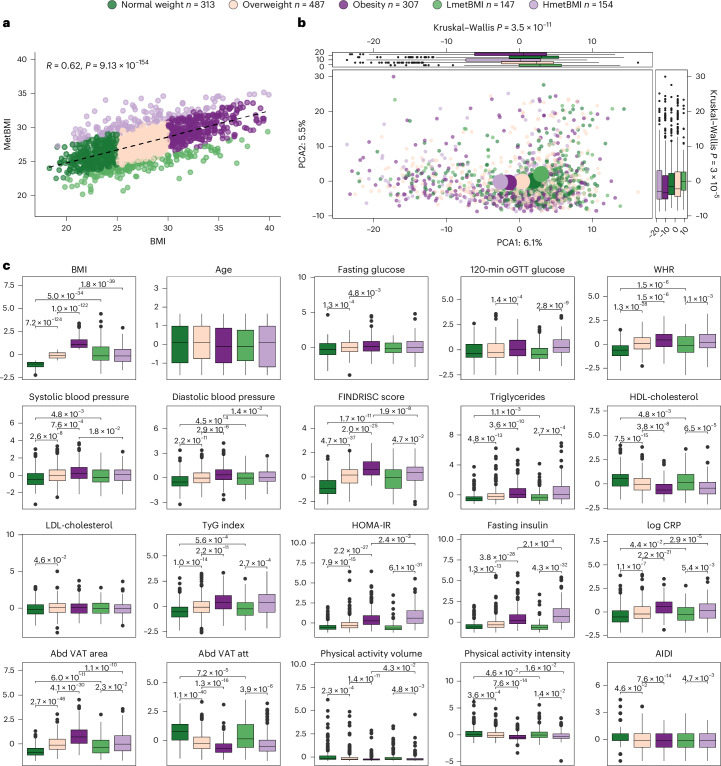
MetBMI corresponds with distinct metabolome entities and clinical phenotypes. **a**, Two-sided Pearson’s correlation between ground truth BMI and metBMI (*n* = 1,408). Each dot represents one individual, colored by metBMI group (sample size per group as described in the legend). Pearson’s coefficient (*r*) and the corresponding *P* value are shown. **b**, Principal component analysis (PCA) of whole plasma metabolome. Each point represents one individual, colored by metBMI group. Large points denote group medoids. Side box plots display metBMI group distributions along PC1 and PC2 (two-sided Kruskal–Wallis derived, *n* = 1,408 and per metBMI group as described in the top legend; *n* for normal weight = 313, overweight = 487, obesity = 307, LmetBMI = 147, HmetBMI = 154). Box plots display the median; interquartile range (IQR) with whiskers specify ±1.5× IQR; and plotted points denote outliers. **c**, Comparisons of *z*-score-transformed anthropometric, metabolic and lifestyle features across metBMI groups (two-sided Kruskal–Wallis tests with Benjamini–Hochberg adjustment). VAT attenuation is shown as absolute values. *n* per group and box plot as in **b**. oGTT, oral glucose tolerance test; FINDRISC, Finnish Diabetes Risk Score; PC, principal component.

**Extended Data Fig. 3:**
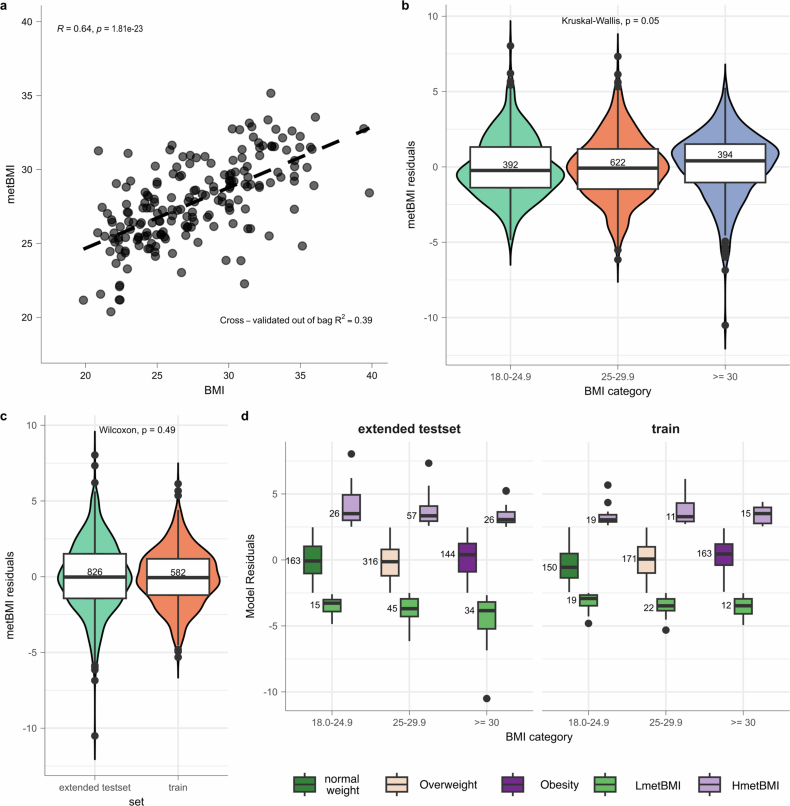
Test diagnostics of the ridge regression model. **a**. two-sided Spearman’s correlation (here denoted as *R*) with its corresponding *P* Value between the ground truth and metabolites-predicted BMI (metBMI) in the IGT-test set (n = 192). The dashed black line is the linear regression line. **b,c** Violin plots of metBMI residuals distributions within BMI-categories in the entire IGT-cohort, *P* is derived from a two-sided Kruskall Wallis test (**b**) and between training and extended test set, the sample size is depicted in the respective boxplot and *P* is derived from a two-sided Wilcoxon rank-sum test (**c**). **d**. Boxplot showing the distribution of metBMI residuals within the IGT cohort, including the training and extended test sets (as in **c**), along with group classifications based on metBMI residuals and WHO BMI categories. Boxplots show the median and interquartile ranges (IQR); whiskers extend to ±1.5*IQR from the quartiles and plotted points denote outliers. Boxplots are labeled with the number of individuals within each of the metBMI-classification group, and colored according to these groups (green for normal weight, taupe for individuals with overweight, purple for individuals with obesity, light green for LmetBMI and light purple for HmetBMI).

To improve the parsimony of the model while addressing colinearity, we trained a ridge regression model using the 267 metabolites most stringently associated with BMI (Methods and Supplementary Table 4). The resulting metBMI was highly correlated with the measured BMI (Fig. 2a; Pearson’s *r* = 0.62, Spearman’s *ρ* = 0.63, *P* < 2.2 × 10^−16^), explaining 39% of BMI variance in the held-out test set of the IGT-microbiota cohort (Extended Data Fig. 3a). Similar results were obtained using least absolute shrinkage and selection operator (LASSO) regression (Methods).

To capture the metabolic signature of obesity across the BMI spectrum, we extracted metBMI residuals for each participant, adjusted for age, sex and BMI. Individuals with disproportionately high (> +2.5) or low (< −2.5) residuals were classified as HmetBMI and LmetBMI, respectively, each representing approximately 10% of the cohort. These groups exhibited distinct metabolomic profiles (*P* = 1.2 × 10^−7^, post hoc Wilcoxon rank-sum test; Fig. 2b). LmetBMI individuals clustered with those of normal weight, whereas HmetBMI individuals clustered with those with obesity, despite similar BMI ranges (range, LmetBMI: 18.98–46.27 kg m^−2^, HmetBMI: 20.59–39.92 kg m^−^^2^, *P* = 0.28, Wilcoxon rank-sum-test; Extended Data Fig. 3b–d) and similar broad clinical characteristics (for example, age, sex, fasting glucose and blood pressure; Fig. 2c).

HmetBMI individuals exhibited hallmarks of metabolic dysfunction, including higher WHR, more severe VAT area and attenuation, elevated triglycerides, insulin resistance (Homeostatic Model Assessment of Insulin Resistance (HOMA-IR)), inflammation (C-reactive protein (CRP)), poorer adherence to an anti-inflammatory diet (Anti-Inflammatory Diet Index (AIDI))^19^ and reduced gut microbiome gene richness compared to LmetBMI (Fig. 2c and Supplementary Table 5). These patterns were consistent across sex and BMI class, highlighting that metBMI captures metabolic risk independent of body size (Supplementary Tables 5 and 6).

Some differences between the HmetBMI and LmetBMI, however, were sex specific: lower physical activity was more pronounced in males, and elevated inflammation and poor adherence to an anti-inflammatory diet were more evident in females (Supplementary Table 6), despite balanced model training and the independence of metBMI residuals from BMI and sex (Methods). Crucially, key discriminators, such as lower gut microbiome gene richness, more pronounced VAT attenuation, insulin resistance and insulin hypersecretion, were consistently observed in HmetBMI across both sexes and BMI classes (Supplementary Tables 5 and 6), emphasizing the unique contribution of hyperinsulinemia, insulin resistance and impaired glucose uptake/utilization in metabolic obesity beyond actual BMI.

**Extended Data Fig. 4:**
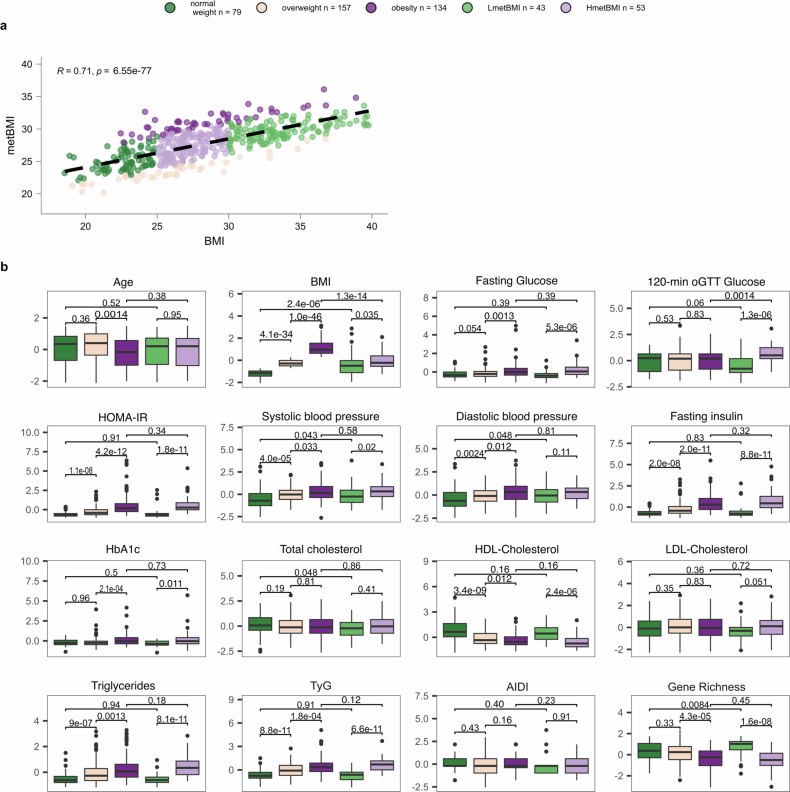
Validation of the metBMI model in the SCAPIS cohort and links to metabolic heterogeneity. **a**. two-sided Spearman’s correlation (denote here as *R*) between ground truth BMI and metabolites-predicted BMI (metBMI). The dashed line is the linear regression line. Each dot represents one individual, colored according to the final metBMI classification group: green for normal weight (n = 79), taupe for individuals with overweight (n = 157), purple for individuals with obesity (n = 134), light green for LmetBMI (n = 43), and light purple for HmetBMI (n = 53). **b**. Comparisons in relevant anthropometric, metabolic, and lifestyle features between the different metBMI groups. Feature values were Z-score transformed prior to plotting, and *P* values are derived from a two-sided Wilcoxon rank-sum test and adjusted for multiple testing, *ad modum* Benjamini-Hochberg, across the five metBMI groups. Boxplots are colored according to these groups and show the median and interquartile ranges (IQR); whiskers extend to ±1.5*IQR from the quartiles and plotted points denote outliers. Sample size per group as in **a**.

These findings were replicated in the independent Swedish Cardiopulmonary Bioimage Study (SCAPIS) cohort (*n* = 466; Supplementary Table 7), where metBMI and BMI remained strongly correlated (*r* = 0.72, *ρ* = 0.71, *P* < 2.2 × 10^−16^, out-of-sample *R*^2^ = 0.52; Extended Data Fig. 4a,b). This cohort had a more balanced sex distribution but was slightly older and showed higher disease burden than the IGT-microbiota cohort. Notably, it included a three-fold higher prevalence of metabolic syndrome, 11% with newly diagnosed T2D at screening and more severe dyslipidemia, despite more intensive treatment with lipid-lowering agents, thus suggesting a further progression of metabolic dysfunction (Supplementary Tables 7 and 8). Within SCAPIS, HmetBMI individuals had slightly higher ground truth BMI than LmetBMI (27.5 kg m^−^^2^ versus 26.2 kg m^−^^2^) but a markedly higher metBMI than the LmetBMI (median 31 kg m^−^^2^ versus 23 kg m^−^^2^) and a more adverse cardiometabolic profile, including elevated triglyceride–glucose (TyG) index and fasting glucose and a higher prevalence of incident T2D (Extended Data Fig. 4b and Supplementary Table 8).

### Clinical risk stratification and intervention response using metBMI and its residuals

**Fig. 3:**
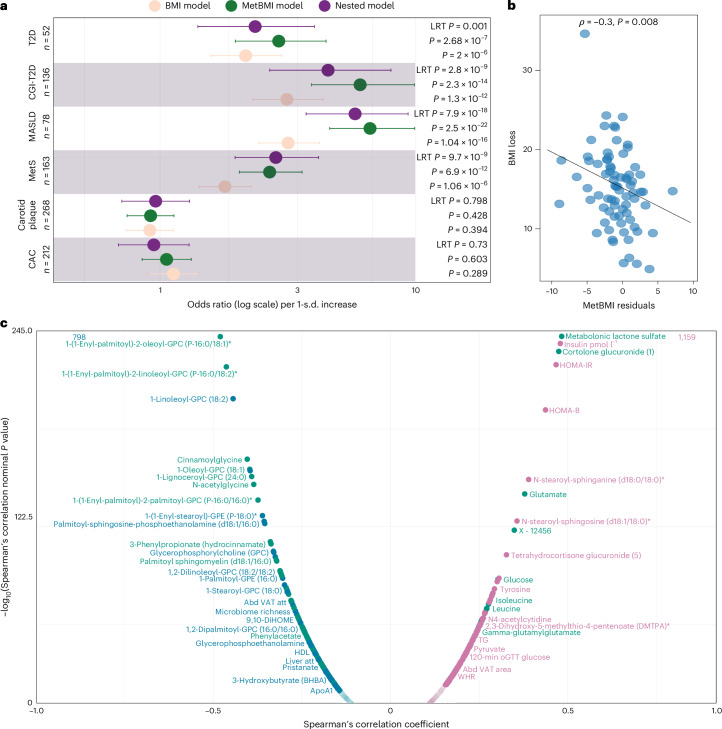
MetBMI and its residuals are associated with higher disease odds, reduced benefit from intervention and consistent molecular phenotypes. **a**, Forest plot for six cross-sectional outcomes in the SCAPIS cohort (CAC score, carotid plaque, MetS, MASLD, CGI-T2D and screen-detected T2D). Data are presented as odds ratio estimated (center points) with 95% confidence intervals (horizontal bars), with lower and higher confidence interval limits from multivariable logistic regression per 1-s.d. increase in the predictor (BMI, metBMI or both in the nested model). The dashed line marks odds ratio = 1. *P* values are derived from two-sided Wald tests for BMI/metBMI. For the nested model, *P* is derived from an LRT versus BMI-only model. Sample sizes per outcome: CAC score (*n* = 212), carotid plaque (*n* = 268), MetS (*n* = 163), MASLD (*n* = 78), CGI-T2D (*n* = 136) and T2D (*n* = 52). **b**, Two-sided Spearmanʼs correlation for metBMI residuals with BMI loss 12 months after bariatric surgery (*n* = 75), with its corresponding *P* value. Each dot represents one individual, and the dashed line represents the linear regression. **c**, Two-sided partial Spearmanʼs correlation between metBMI residuals and all available circulating metabolites, proteins and clinical chemistry, corrected for age, sex and BMI in the IGT-microbiota cohort (*n* = 1,408). Positive correlations are in pink; negative correlations are in blue. Metabolites with variance explained >20% (ref. ^32^) or predominantly predicted by the microbiome^11^ are highlighted in green. Only Benjamini–Hochberg-adjusted significant correlations are shown (*q* < 0.05). ApoA1, apolipoprotein A1; TG, triglycerides.

To evaluate the predictive utility of metBMI, we tested its ability to classify six cardiometabolic outcomes in the SCAPIS cohort using logistic regression adjusted for age and sex (Methods). For each outcome, we compared three models: one with BMI, one with metBMI and a nested model including both. Likelihood ratio tests (LRTs) assessed whether metBMI added explanatory power beyond BMI in the nested model. MetBMI yielded the strongest predictive performance for metabolic syndrome (MetS), metabolic dysfunction-associated steatotic liver disease (MASLD), combined impaired fasting and postprandial glucose (Combined Glucose Intolerance and Type 2 Diabetes (CGI-T2D)) and screen-detected T2D (Fig. 3a). In metBMI-only models, the predicted odds ratios per 1-s.d. metBMI increase were substantial and statistically significant (MetS: odds ratio = 5.36 (95% confidence interval: 3.88–7.66, *P* = 2.6 × 10^−22^); MASLD: odds ratio = 4.95 (95% confidence interval: 3.36–7.65, *P* = 2.3 × 10^−14^); CGI-T2D: odds ratio = 2.40 (95% confidence interval: 1.88–3.11, *P* = 6.9 × 10^−12^); screen-detected T2D: odds ratio = 2.6 (95% confidence interval: 1.83–3.77, *P* = 2.7 × 10^−7^)). Nested models demonstrated a significantly improved fit compared to BMI alone (Fig. 3a), suggesting that metBMI captures additional disease signals. However, neither BMI nor metBMI predicted subclinical atherosclerosis (Coronary Artery Calcium (CAC) score and carotid plaque presence; *P* > 0.3 for LRTs).

**Extended Data Fig. 5:**
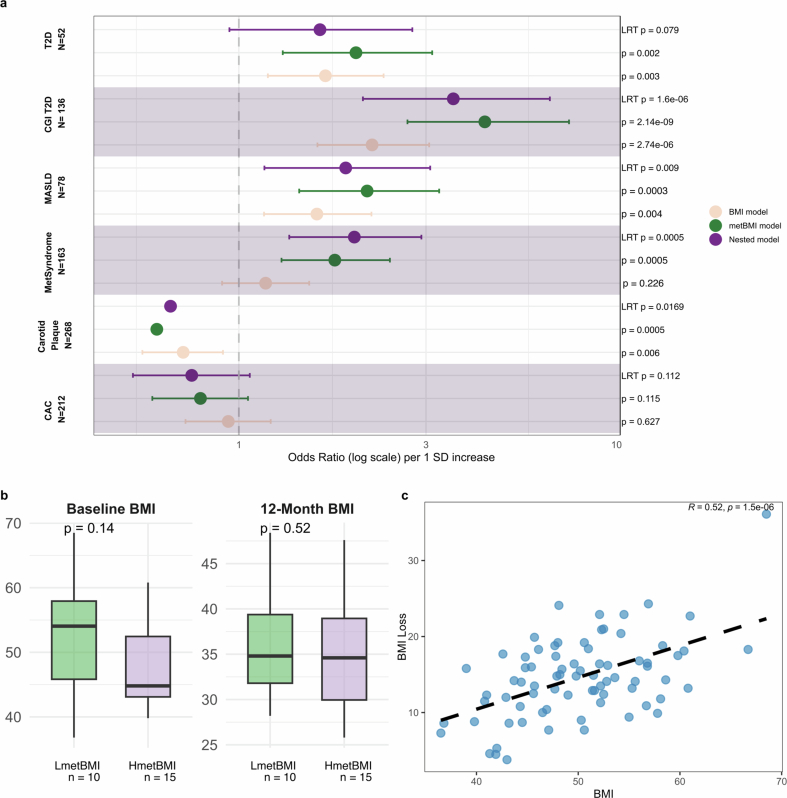
Metabolomics-derived BMI in cardiometabolic risk and outcome. **a**. Forest plot shows adjusted odds ratios (ORs) and 95 % confidence intervals for six cross-sectional outcomes in the SCAPIS cohort: coronary artery calcium (CAC), carotid plaque, metabolic syndrome (MetS; NCEP criteria), non-alcoholic fatty liver disease (NAFLD), combined glucose intolerance + newly detected type 2 diabetes (CGI T2D) and screen-detected T2D (sample sizes, left). Data are presented as odds ratio estimated (centre points) with 95% confidence intervals (horizontal bars) with lower and higher confidence interval limits from multivariable logistic regression per 1 SD increase in the predictor (BMI, metBMI or both in the nested model) and plotted on a log10 scale. Models adjust for age, sex, waist-to-hip ratio, HDL, LDL, and total triglyceride levels, mean systolic and diastolic blood pressure, glucose levels, and statin use. The dashed line marks OR = 1. *P* values are derived from two-sided Wald tests for BMI/metBMI. For the nested model, *P* is derived from a likelihood-ratio test vs BMI-only model. Sample sizes per outcome; CAC (n = 212), carotid plaque (n = 268), metabolic syndrome (n = 163), MASLD (n = 78), CGI/T2D (n = 136), T2D (n = 52). **b**. boxplots depicting differences in baseline BMI, and 12-month BMI in bariatric surgery (n = 75) between LmetBMI and HmetBMI. *P* values for the two-sided Wilcoxon-rank-sum test are displayed, and sample sizes for each group are shown. Boxplots show the median and interquartile ranges (IQR); whiskers extend to ±1.5*IQR from the quartiles and plotted points denote outliers. **c**. Two-sided Pearson’s correlation between ground-truth BMI at baseline and BMI loss at 12 months after bariatric surgery (n = 75), with the corresponding *P* value. The dashed line is the linear regression line.

The associations remained robust after adjusting for traditional risk factors (lipids, glucose, blood pressure, WHR and statin use). MetBMI remained a strong and independent predictor of MetS (odds ratio = 2.12, 95% confidence interval: 1.43–3.24, *P* = 3.1 × 10^−4^), MASLD (odds ratio = 4.24, 95% confidence interval: 2.69–6.95, *P* = 2.1 × 10^−9^) and CGI-T2D (odds ratio = 1.76, 95% confidence interval: 1.28–2.43, *P* = 5.0 × 10^−4^) risk (Extended Data Fig. 5a); continued to add predictive value over BMI in nested models for MetS (LRT *P* = 0.0005) and CGI-T2D (LRT *P* = 1.6 × 10^−6^); and, unexpectedly, reduced carotid plaque burden (LRT *P* = 0.017) (Extended Data Fig. 5a).

In an independent bariatric surgery cohort^20^ (*n* = 75; Methods), baseline metBMI residuals were inversely correlated with BMI loss/reduction at 12 months (*r* = −0.30, *P* = 0.008; Fig. 3b), despite no significant difference in baseline or follow-up BMI between HmetBMI and LmetBMI (Extended Data Fig. 5b). As expected, a higher BMI was associated with greater absolute BMI loss (Extended Data Fig. 5c). These findings highlight a dissociation between BMI and metBMI: whereas higher BMI predicts greater weight loss, higher metBMI residuals predict poorer response, suggesting that metBMI captures aspects of metabolic resistance to intervention that are not reflected in BMI alone.

Together, these findings establish metBMI and its residuals as biomarkers of a metabolically adverse obesogenic signature, capturing risk and intervention response beyond BMI and other traditional risk factors.

### Characterizing clinical and multi-omics signatures of metBMI residuals

**Extended Data Fig. 6:**
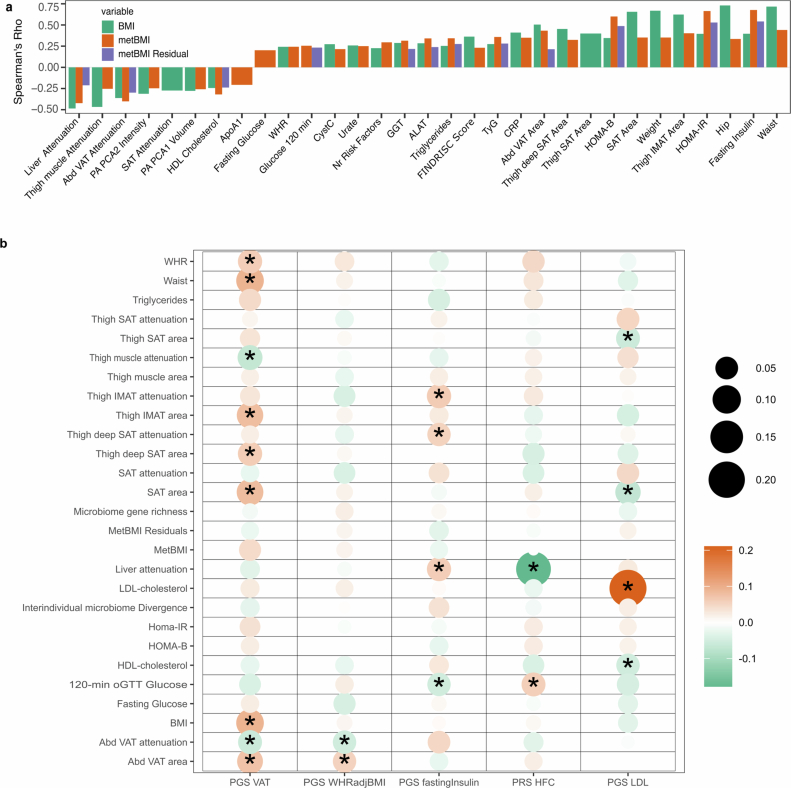
Correlation analysis for metBMI and metBMI residuals with metabolic, anthropometric, and polygenic risk score. **a**. Two-sided partial Spearman’s rank correlations of available metadata, corrected for age, sex, and BMI for metBMI and metBMI residuals, and for age and sex for BMI in the IGT-microbiota cohort (n = 1,408). Only correlations significant after multiple testing correction *ad modum* Benjamini-Hochberg (*Q* values < 0.05) and with absolute correlation coefficients > 0.2 are shown. Colors indicate the correlation with the specific obesity metric: orange for metBMI, green for BMI, and purple for metBMI residuals. Corresponding values are found under Supplementary Table 9**. b**. Two-sided Spearman’s rank correlation of calculated PGS using weighted effect alleles with their predicted and other anthropometric and metabolic traits, metBMI and metBMI residuals, as well as microbiome features such as microbiome gene richness and divergence (average sample dissimilarity based on Bray-Curtis distance) the IGT-microbiota cohort (n = 1,408). Signs and weights of effect sizes are indicated by marker color and size of the circle, as shown in the legend. Significant correlations are denoted with an Asterisk. ***Abbreviations****:* WHR (waist-to-hip ratio), SAT (subcutaneous adipose tissue), IMAT (intramuscular adipose tissue), LDL (low-density lipoprotein), HOMA-IR (Homeostatic Model Assessment of Insulin Resistance), VAT (visceral adipose tissue area), HOMA-B (Homeostatic Model Assessment of Beta-cell function), HDL (high-density lipoprotein), BMI (body mass index), [Abd] VAT ([abdominal] visceral adipose tissue), PGS (polygenic risk score), WHRadjBMI (WHR adjusted for BMI), HFC (hepatic fat content).

Next, we assessed how metBMI residuals relate to metabolic, anthropometric and omics data to identify the biological features behind the metabolic obesogenic signature. These residuals, orthogonal to BMI, age and sex, correlated more strongly with VAT attenuation, an imaging proxy for adipose tissue lipid content and fibrosis^21^, than with VAT area or liver attenuation, both indicators of ectopic fat. Additionally, metBMI residuals correlated more strongly than BMI with insulin resistance, β-cell-linked insulin hypersecretion (Homeostatic Model Assessment of β cell function (HOMA-B), fasting insulin) and impaired glucose tolerance (Extended Data Fig. 6a). Mediation analysis revealed that metBMI residuals mediated 38% of the effects of VAT attenuation (that is, adipose tissue architecture) on β cell function (HOMA-B; bootstrap 95% confidence interval: 0.28–0.51, *P* < 2 × 10^−16^), supporting their role in inter-organ metabolic regulation.

In line with these results, metBMI residuals positively associated with steroidal metabolites implicated in insulin resistance and cardiometabolic disease (for example, metabolomic lactone sulfate^22^ and cortolone glucuronide) as well as with glutamate and inversely with glutamine. The balance between these two amino acids, previously identified as a marker of adipose tissue dysfunction^23^, is highly predicted by the microbiome in our cohort (Supplementary Table 3). Other metabolites positively associated with metBMI residuals included branched-chain and aromatic amino acids as well as several phosphoinositol and phosphatidylethanolamine species. Inverse correlations included phosphatidylcholines, acetyl-carnitines, gut and diet-derived carotene diols and cinnamoylglycine^11^ (Fig. 3c and Supplementary Table 9).

MetBMI residuals were also associated with proteome features involved in insulin responsiveness and energy regulation across central, hepatic and adipose tissues. Positively correlated proteins included oxytocin, carboxylesterase 1 (ref. ^24^), leptin^25^ and asialoglycoprotein receptor 1, the latter reported to impair hepatic cholesterol clearance, thereby elevating circulating lipids^26^. In agreement, metBMI residuals were inversely correlated with insulin-like growth factor binding protein 2, whose deficiency exacerbates hepatic steatosis and worsens MASLD phenotypes^27^.

To assess heritability, we tested polygenic risk scores (PRSs) related to insulin secretion, adipose tissue distribution, circulating lipids and ectopic fat accumulation^28–30^: although each PRS correlated with its respective trait, neither metBMI nor its residuals was significantly captured by any PRS (Extended Data Fig. 6b).

These findings indicate that metBMI residuals reflect a non-genetic, acquired metabolic signature characterized by ectopic fat accumulation, hepatic and adipose tissue dysfunction and altered insulin signaling across omics. This aligns with the Twin Cycle Hypothesis^31^, whereby, depending on a personal fat threshold, liver and pancreatic interactions contribute to the individual pathogenesis of insulin resistance and metabolic disease, independent of BMI-defined obesity and across the entire BMI range.

### Microbiome features of the obesogenic signature

**Fig. 4:**
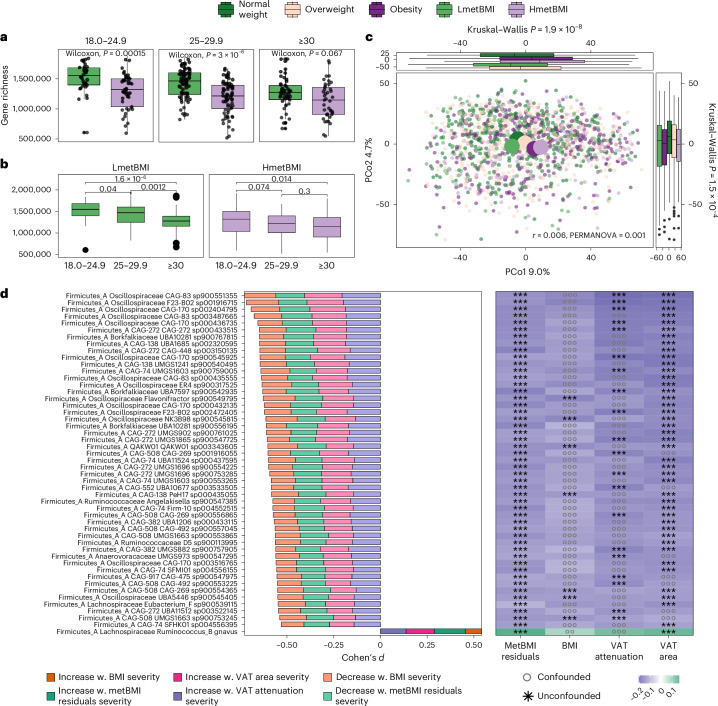
MetBMI groups correspond to distinct gut microbiome states and have shared species with other obesity measures. **a**,**b**, Microbial gene richness for individuals with lower and higher predicted metBMI within BMI classes (**a**) and metBMI groups across BMI classes (**b**), assessed using two-sided Wilcoxon rank-sum tests. Sample sizes: BMI 18.0–24.9 kg m^−^^2^: LmetBMI *n* = 34, HmetBMI *n* = 45; BMI 25–29.9 kg m^−^^2^: LmetBMI *n* = 67, HmetBMI *n* = 68; BMI ≥ 30 kg m^−^^2^: LmetBMI *n* = 46, HmetBMI *n* = 41. **c**, PCoA of gut microbial communities (Aitchison distance) in the IGT cohort (*n* = 1,408), colored by metBMI group: green, normal weight (*n* = 313); taupe, overweight (*n* = 487); purple, obesity (*n* = 307); light green, LmetBMI (*n* = 147); light purple, HmetBMI (*n* = 154). Large dots indicate group medoids. Variance explained by metBMI group and *P* values from one-sided PERMANOVA are shown. Side box plots depict group distributions across the first and second principal coordinates (two-sided Kruskal–Wallis test). In **a**–**c**, box plots show median (center line), IQR (box), whiskers to the most extreme points within 1.5× IQR and outliers as points. **d**, Top 50 differentially abundant bacterial species overlapping in all obesity measures. Left: feature contributions to effect size (darker = increase). Right: associations with obesity measures, adjusted for other measures; signed effect size indicated by marker color (green, increased; violet, decreased). Asterisks mark features not confounded by other measures; circles indicate confounded features. **q < 0.01; ***q < 0.001. Full data are in Supplementary Table 12. W., with.

**Extended Data Fig. 7:**
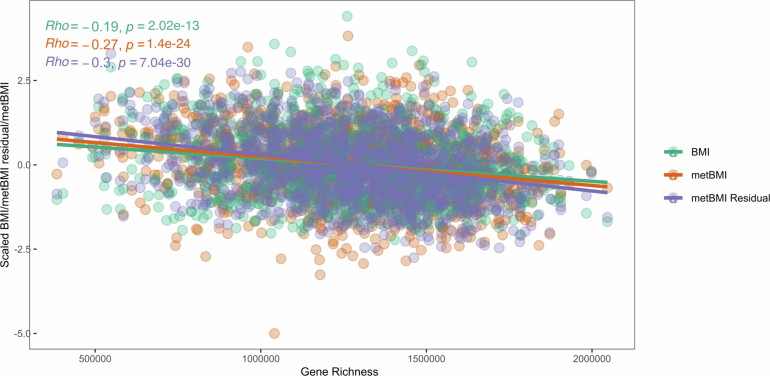
Microbiome gene richness along BMI, metBMI and metBMI residual spectrum. Two-sided Spearman’s correlation between Z-transformed gound-truth BMI, metBMI, and metBMI residuals with gut microbiome gene richness in the IGT-microbiota cohort. The lines correspond to the linear regression lines colored according to the relevant correlated variable (green for BMI, purple for metBMI residuals, and orange for metBMI). Relevant correlation coefficients and *P* values are denoted and colored according to the specific variable in the legend, top left. *Rho* depicts Spearman’s ranked correlation coefficient.

Given the links between host metabolism and the gut microbiome^15,17^, we examined how metBMI and its residuals relate to gut microbiome diversity, ecological structure, composition and function. MetBMI and its residuals were more strongly and negatively correlated with gene richness than BMI (*ρ* = −0.19, −0.24 and −0.3 for BMI, metBMI residuals and metBMI, respectively; *P* < 2.2 × 10^−16^ for all correlations and false discovery rate (FDR) < 0.05, adjusted for age and sex as well as BMI where appropriate; Extended Data Fig. 7). In multivariable models, the addition of metBMI eliminated the significant correlation of gene richness and 359 metabolic, dietary and inflammatory markers, including BMI, HOMA-IR, MetS, WHR, CRP, renal function, leptin and dietary variables (Supplementary Table 10), highlighting metBMI as a concise summary of inter-organ and inter-organismal interactions. Notably, the gene richness of individuals with normal weight but high residuals (HmetBMI) was as low as that of individuals with obesity in the LmetBMI group (*P* = 0.06; Fig. 4a,b), indicating that erosion of microbiome diversity accelerates with metabolically adverse adiposity.

**Extended Data Fig. 8:**
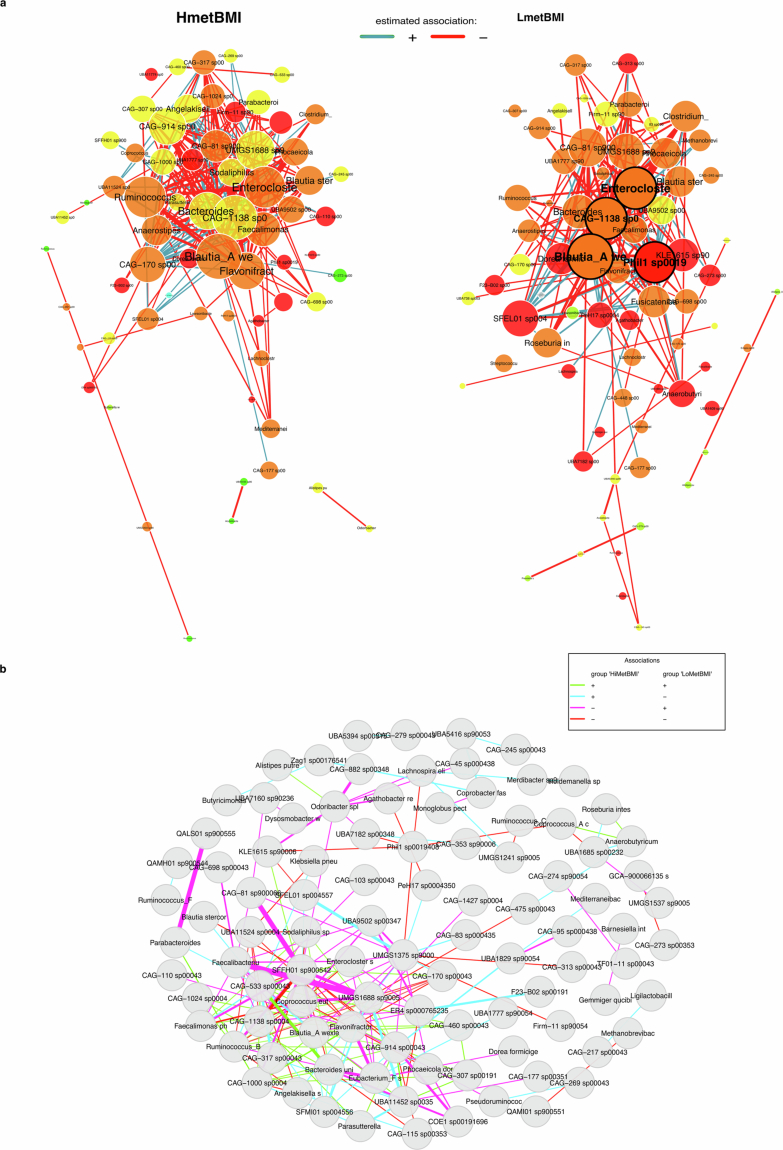
Bacterial network topology in HmetBMI and LmetBMI and altered global influence of specific taxa in the network structure of HmetBMI and LmetBMI. **a**. Species-level bacterial association networks for high (HmetBMI) and low (LmetBMI) metabolic BMI groups, based on two-sided Spearman’s correlations of CLR-transformed abundances (sparsification threshold ≥ 0.3). Analysis was restricted to the 500 most variable species, with zeros imputed via multiplicative simple replacement. Node size reflects eigenvector centrality; node color indicates clusters defined by greedy modularity optimization. Blue and red edges denote positive and negative associations, respectively. The Layout from the HmetBMI network was applied to both groups, with unconnected nodes omitted for a better overview. **b**. Differential association networks showing connected nodes if they are differentially associated between HmetBMI and LmetBMI. The Fisher’s Z-test is applied to identify differentially correlated taxa. Multiple testing adjustment is performed by controlling the local false discovery rate. Shown are two-sided Spearman’s rank correlations after clr (centered log-ratio) transformation of abundances on species levels, applying a sparsification threshold of 0.3 (only absolute correlations ≥0.3 are retained). The analysis included the 500 species with the highest variance, and zeros were replaced using the multiplicative simple replacement method. Edge colors represent the direction of the associations in the two groups as indicated in the legend.

Beyond gene richness, HmetBMI and LmetBMI groups exhibited distinct microbiome community structures. Principal coordinate analysis (PCoA) revealed clear compositional separation and clustering of HmetBMI with obesity and LmetBMI with normal weight (Fig. 4c), consistent with the observed metabolome patterns (Fig. 2b). These differences extended to ecological order, as indicated by network analyses. We observed low similarity between the two clusterings and denser, more modular consortia in LmetBMI, with a greater degree of eigenvector centrality (*P* = 0.000009 and *P* = 0.0000081, respectively, adjusted Rand index = 0.0001), indicating a larger number of interactions between nodes, anchored by Christensenellales (for example, *Phil1 sp001940855*) and *Methanobrevibacter smithii* (Extended Data Fig. 8a and Supplementary Table 11). HmetBMI networks were sparser and centered around taxa linked to metabolic dysfunction (for example, *Blautia*, *Bacteroides*, *Flavonifractor*, *Erysipeloclostridium ramosum* and *Ruminococcus gnavus*), which exhibited more negative interactions with health-related taxa, such as *Faecalibacterium* and *Eubacterium* (Extended Data Fig. 8b and Supplementary Table 11).

**Extended Data Fig. 9:**
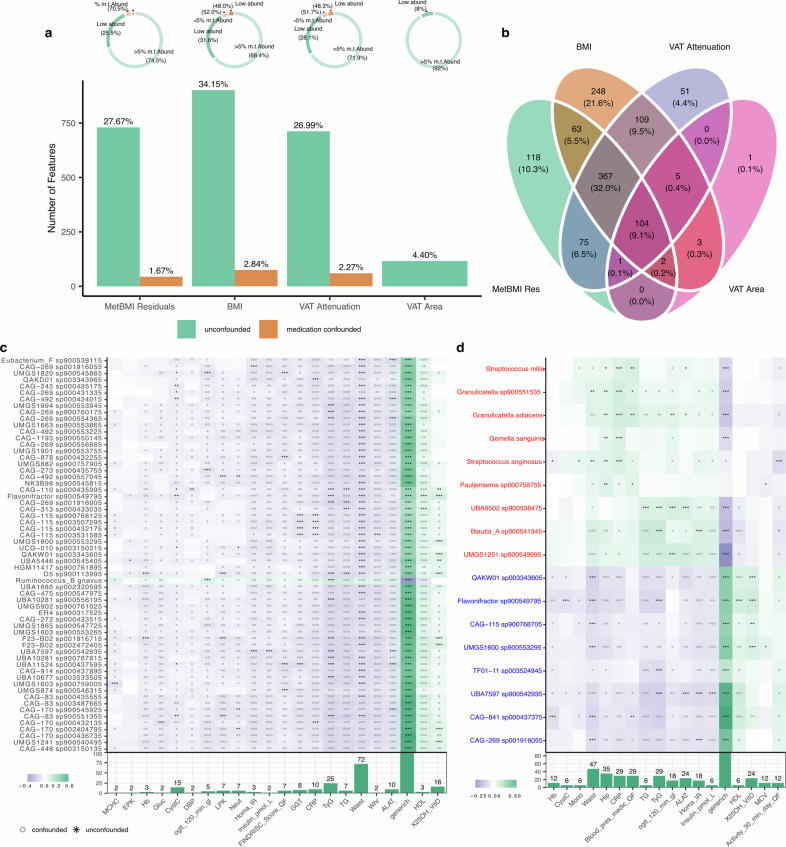
Shared microbiome features of obesity measures and their metabolic associations. **a**. Barplots show number of differentially abundant taxa across metBMI residuals, BMI, VAT attenuation, and area, categorized as confounded (orange) or unconfounded (green) by medication. Circles above bars indicate proportions of low-abundant features (see methods, shaded by confounding status). Low-abundant species constituted 33%, 31%, and 8% of taxa along BMI, VAT attenuation, and VAT area, respectively, with ~ 50% confounded by medication (except VAT area). **b**. Venn Diagram illustrates taxa-overlaps between traits (ANCOM-BC, Cohen’s D > 0.05, *Q* value < 0.1). **c**. Heatmap displays two-sided Spearman’s correlations between shared species and clinical markers (*Q* value < 0.1) in the IGT-microbiota cohort (n = 1,408). Marker color indicates effect direction. Bar plots show the percentage of features in the heatmap significantly correlated with the covariate. **d**. Similar to **c**, showing taxa uniquely associated with metBMI residuals. ** *P* value < 0.01, *** *P* value < 0.001.

Species-level modeling, adjusted for medication and mutually controlling for BMI, VAT area and attenuation, identified 774 taxa associated with metBMI residuals (Supplementary Table 12 and Extended Data Fig. 9a,b). Of the 104 species shared with other adiposity metrics, 100 were primarily driven by metBMI residuals (Fig. 4d), with *R. gnavus* being the only species enriched across all traits and correlated with impaired glucose tolerance and the TyG index (Fig. 4d and Extended Data Fig. 9c). To exclude that changes in microbiome composition at the species level were secondary to decreasing microbiome richness, we adjusted for the latter. We observed that 45 taxa remained significantly associated with metBMI residuals, most notably *Faecalibacterium prausnitzii* and Oscillospiraceae (decreased) and oral/aerotolerant species (*Streptococcus anginosus*, *Streptococcus mitis*, *Gemella* and *Granulicatella*), which increased with metBMI residuals (Extended Data Fig. 9d). These species associated with low-grade inflammation and shifts in fatty acid, bile acid and environmental exposures, such as the plasticizer methyladipate (Supplementary Table 13). Although oral taxa tracked with proton pump inhibitor (PPI) levels, their enrichment with increasing residuals was independent of medication, suggesting parallel ecological changes created by drugs^18^ and metabolic injury.

Functionally, 57 GMMs associated with metBMI residuals independently of BMI or other adiposity traits (Supplementary Table 14). Residuals were marked by reduced butyrate production, mannose/glycerol utilization and increased trimethylamine production from γ-butyrobetaine and methanogenesis from trimethylamine. Even after adjusting for gene richness, two hydrogenotrophic processes remained significant along metBMI residuals—decreased methanogenesis from carbon dioxide and increased homoacetogenesis—indicating a shift in microbial carbon dioxide and hydrogen utilization, converted to acetate in HmetBMI or dissipated to methane in LmetBMI.

Together, these data suggest that metBMI residuals reflect a microbiome signature characterized by reduced diversity, altered network structure and functional shifts toward pro-inflammatory and atherogenesis-associated metabolism, capturing aspects of metabolic disruption not explained by BMI alone.

### Metabolite-mediated microbiome–phenotype interactions

Gut bacteria substantially influence the circulating metabolome^11^, as also seen in our study (26% of inter-individual metabolite variance explained by MAGs in median; Supplementary Table 3 and Extended Data Fig. 2c) and in SCAPIS (27% variance explained)^32^. Given the strong covariance in metabolome and microbiome compositions, we postulated that metabolites driving the underlying metBMI signature might be closely related to the microbiome. We generated a clinically tractable signature by applying recursive feature elimination (RFE) and LASSO across 10 resamples, retaining 66 metabolites that best captured metBMI residuals (Supplementary Table 15). This reduced panel explained 38.6% of BMI variance, similar to the performance of the full 267-metabolite model (40%) and markedly more than a model comprising age, sex, triglycerides, high-density lipoprotein (HDL), low-density lipoprotein (LDL), total cholesterol and insulin (26%).

**Fig. 5:**
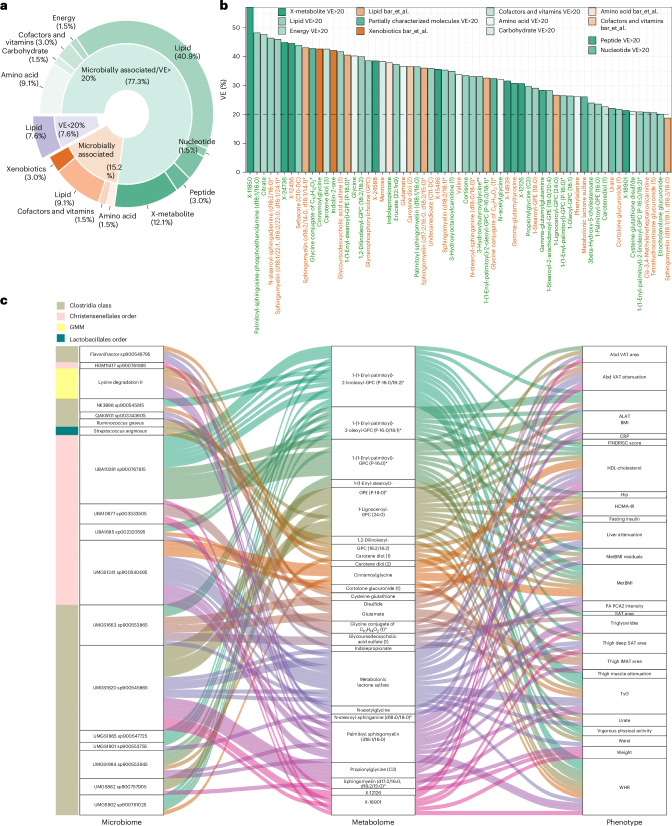
Metabolite predictions by the microbiome and mediation analyses reveal linkages among bacterial species, metabolites and host phenotypes. **a**, Donut plot showing microbially determined metabolites^11^ (orange) and metabolites with more than 20% variance explained (green), across our cohort and external cohorts^32^. Superpathways of these metabolites are displayed above and labeled with their proportions relative to all measured metabolites. **b**, Bar plot showing the median variance explained (%VE in ten models) by bacterial species for the top predictive metabolites of metBMI. Metabolite labels are colored by direction of effect on metBMI (green for lower metBMI, orange for higher metBMI). Bar colors denote superpathways. The horizontal dashed line marks the 20% VE threshold^32^. **c**, Alluvial plot of significant mediation paths (*q* < 0.05) between microbiome features (left) and phenotypes (right) via metabolites (middle), excluding reverse mediations. Curved lines indicate mediation effects, colored according to microbiome features. Left-side bars indicate taxonomic or functional group membership. ALAT, alanine aminotransferase; IMAT, intermuscular adipose tissue; PA, physical activity.

**Extended Data Fig. 10:**
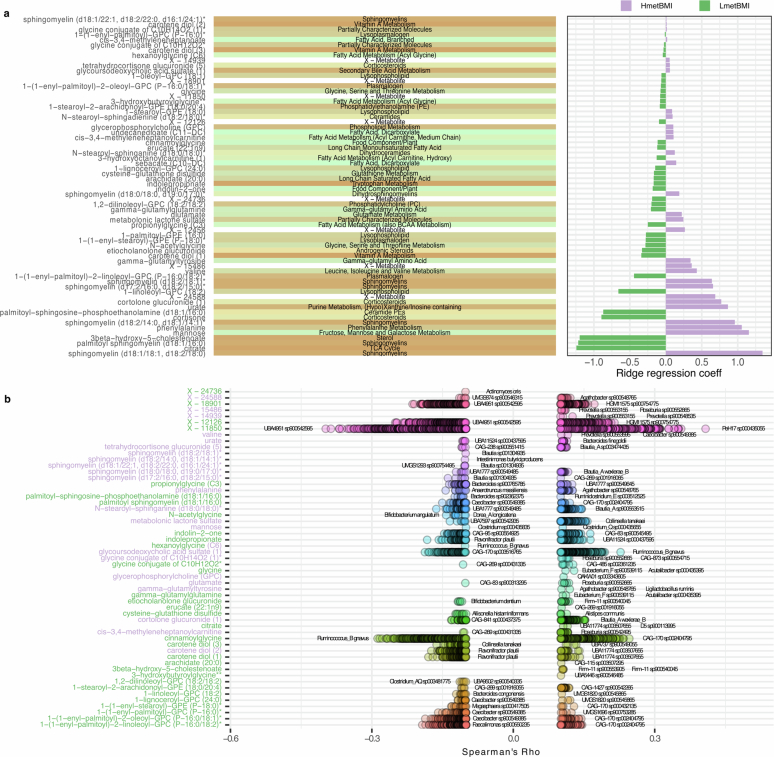
Top metabolites predictive of metBMI show wide associations with microbiome species. **a**. Contribution of top predictor metabolites (selected by recursive feature elimination or retained across all 10 LASSO models) to the model. Signed effect sizes are depicted by color (purple for higher predicted metBMI with positive coefficients, green for lower predicted metBMI with negative coefficients). Metabolites are labeled and colored by subpathway, with each subpathway represented by a unique tile color. **b**. two-sided Spearman’s correlations between 708 gut microbial species and 55 metabolites from the top 66 metabolites selected in recursive feature elimination and all 10 LASSO models (shown in **a**) in the IGT-microbiota cohort (n = 1,408). Displayed are Spearman’s Rho values for significant associations after adjusting for multiple testing *ad modum* Benjamini–Hochberg (*Q* value < 0.05). Metabolite labels are colored based on their effect direction in metBMI prediction (purple for higher metBMI, green for lower metBMI).

For 61 of 66 metabolites, microbial species accounted for more variance than diet or host genetics (FDR < 0.05; Fig. 5a,b and Supplementary Table 15). Of these, metabolites enriched with metBMI residuals included multiple sphingomyelins, ceramides and the microbial fatty acid derivative *cis*-3,4-methyleneheptanoylcarnitine, previously linked to insulin resistance and T2D^33^. Conversely, lower metBMI residuals were associated with 3β-hydroxy-5-cholestenoate, N-acetylglycine, indolepropionate and carotene diols, the latter two being diet-dependent bacterial metabolites with protective effects against cardiovascular risk and T2D^34,35^ (Fig. 5b, Extended Data Fig. 10a and Supplementary Table 15). Building on the correlations between bacterial species specific to metBMI residuals and the selected metabolites (absolute *ρ* > 0.1, FDR < 0.05; Extended Data Fig. 10b), we explored how bacteria may influence host phenotypes by conducting bidirectional mediation analyses among microbiome species, metabolites and clinical traits.

Among the 116 microbiome-to-phenotype pathways mediated by metabolites, bacteria from the Oscillospiraceae family (for example, uncharacterized taxa in *NK3B98*, *UMGS902* and *UMGS1865*) and Christensenellales exerted protective effects via anti-inflammatory and lipid-based metabolites. For example, 1-(1-enyl-palmitoyl)-2-linoleoyl-GPC (P-16:0/18:2)^36^ mediated the impact of Oscillospiraceae on VAT attenuation, improved circulating lipid profiles and lower metBMI. Similarly, cinnamoglycine, a metabolite associated with microbial diversity^15^, carotene diols and palmitoyl sphingomyelin (d18:1/16:0), connected several Clostridia species, Christensenellales and the lysine degradation pathway of the microbiome, involved in butyrate production, with reduced WHR, improved insulin sensitivity and lower liver fat (Fig. 5c and Supplementary Tables 16–18). By contrast, bacterial species linked to higher adiposity markers and metBMI residuals, such as *R. gnavus* and aerotolerant/oral bacteria, exerted effects through depletion of these protective metabolites, reported reduced with escalating cardiometabolic and vascular disease^17^ (Fig. 5c).

Notably, 186 reverse linkages (phenotype-to-microbiome) were identified, implicating systemic inflammation (for example, CRP), dietary vitamin B6 and lipid traits in shaping microbial functions. These effects were direct (147 linkages), mediated by metabolites (seven linkages) or a combination of both (32 linkages) and were associated with functional shifts, including increased triacylglycerol and glutamine degradation and reduced dissimilatory nitrate reduction (Supplementary Table 18).

**Fig. 6:**
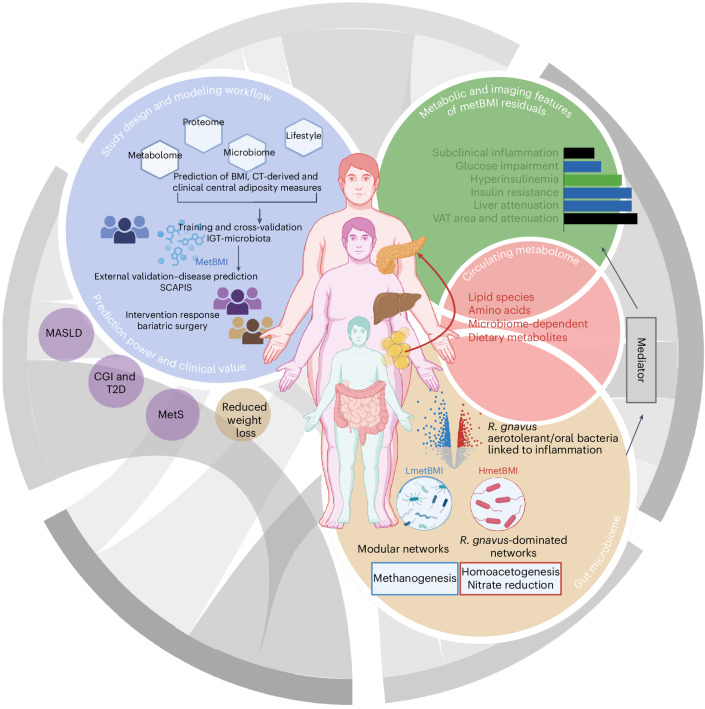
Systems view of metabolic obesity: integrating multi-organ and multi-omics signatures. Light blue circle: deep phenotyping in the IGT-microbiota cohort (*n* = 1,408), including metabolomics, proteomics, metagenomics, diet and clinical profiling, enabled development of metBMI using ridge regression. MetBMI outperformed other omics-based and multi-omics models in capturing central adiposity, explaining over 50% of BMI variance in an external cohort (*n* = 466). In a surgical cohort (*n* = 75), higher metBMI residuals, adjusted for age, sex and BMI, were associated with approximately 30% less weight loss after 1 year. Light green circle: metBMI residuals identified individuals with metabolically adverse obesity, marked by greater VAT area and more severe attenuation, and mediated the relationship between adipose tissue characteristics and insulin hypersecretion. Light taupe circle: these residuals were linked to reduced gut microbial gene richness, altered ecological networks and enrichment of *R. gnavus* and aerotolerant/oral bacteria. Functional shifts included increased nitrate respiration and homoacetogenesis, alongside a reduction in methanogenesis. Light red circle: recursive feature selection and bidirectional mediation analyses identified 116 microbiome → phenotype and 186 phenotype → microbiome paths, primarily mediated by 66 circulating metabolites. This reveals a bidirectional, metabolite-centered interface between the gut microbiome and host metabolism, providing insights into the heterogeneity of obesity and its clinical manifestations. Figure created with BioRender.com. CT, computed tomography.

These findings demonstrate that metBMI residuals capture a bidirectional host–microbiome axis, suggesting that circulating metabolites may not only serve as functional proxies for microbiome composition but also mediate the effects of bacterial species on metabolic risk phenotypes. Disruptions in these microbiome–metabolome interactions may contribute to the metabolic dysfunction observed in subclinical adiposity-driven changes along the BMI spectrum, independent of obesity-defining thresholds (Fig. 6). This putative mechanistic link also explains the superior risk stratification of metBMI over BMI.

## Discussion

In this study, we demonstrate that metBMI and its residuals capture the metabolic signature of obesity across the BMI spectrum. MetBMI outperforms other omics-derived BMI models in aligning with contemporary definitions of obesity^2^, emphasizing central adiposity over conventional BMI thresholds. MetBMI residuals provide a refined measure of metabolic burden, independent of measured BMI, yet strongly linked to visceral fat distribution, insulin resistance and hypersecretion, impaired glucose tolerance and increased cardiometabolic risk for T2D and fatty liver disease, consistent with the Twin Cycle Hypothesis^31^ and recent reports linking metabolically predicted BMI to elevated T2D morbidity and mortality^9^.

Our metBMI also compares favorably with previous efforts. Cirulli et al.^8^ used 650 metabolites to explain approximately 50% of BMI variance, retaining 43% explanatory power with 49 metabolites without external validation. Gerl et al.^37^ reported 47% variance explained using 75 lipidomic features (with age and sex included), whereas Beyene et al.^38^ achieved 52% in external validation using 575 lipid species. Watanabe et al.^10^ reported *R*^2^ of 0.7 internally but only 0.3 in external validation. Although their metabolite-based BMI decreased after intervention (as opposed to protein-predicted BMI), its predictive value for outcomes was not assessed. In this context, our 66-metabolite signature retains 38.6% of the 40% explanatory power observed for the full 267-metabolite model, and residuals were linked to poorer post-surgical weight loss, underscoring the model’s clinical utility. Discriminative metabolites in our model, including branched-chain amino acids, long-chain fatty acids and phospholipids, have been associated with higher BMI predictions in large cohorts^8–10^, and several have been mechanistically linked to insulin resistance and T2D^39^, underscoring robustness in our findings.

Detailed phenotyping in the IGT-microbiota cohort identified VAT as a key driver of metBMI. Notably, metBMI residuals correlated with VAT area and even more strongly with VAT attenuation, a computed tomography-derived proxy for adipocyte hypertrophy, mirroring findings that multi-omics-derived BMI is influenced by adipokines such as leptin^10^, a hormone associated with adipocyte size^25^, VAT attenuation and increased cardiovascular risk^21^.

A still-underexplored dimension of obesity’s metabolic heterogeneity is its relationship with the gut microbiome and its extensive metabolic capacity^14^. MetBMI was robustly captured by microbiome composition, and several signature metabolites were microbially produced or highly predictable from microbial features. For instance, cinnamoylglycine mediated potentially causal microbiome links to reduced WHR, improved insulin sensitivity and lower liver fat. Elevated metBMI was associated with microbial networks of reduced connectivity and modularity, suggesting a greater susceptibility to environmental influences, alongside decreased fermentative activity, increased potential for anaerobic respiration (for example, nitrate reduction) and altered methanogenesis patterns. These shifts have been linked to gut inflammation and ectopic oral bacterial colonization^40^. Reduced methanogenesis from carbon dioxide, on the other hand, with compensatory trimethylamine and increased trimethylamine production potential may promote trimethylamine N-oxide generation by the host and heighten cardiovascular risk^14^. Concomitantly, the increased potential for homoacetogenesis (that is, reductive acetogenesis from carbon dioxide and hydrogen scavenging under conditions of impaired methanogenesis) may elevate acetate availability, promoting hepatic lipogenesis^41^. These findings align with previous studies associating enhanced methanogenic potential with leanness and improved metabolic health^16,42^.

In the altered gut microbial ecology associated with HmetBMI, *R. gnavus* abundance was increased despite a stable prevalence across individuals, tracking closely with VAT area, consistent with previous studies^43^, and associating with insulin resistance and cardiovascular risk, independent of gene richness. This may implicate *R. gnavus* in metabolic dysfunction via tryptophan^44^ and bile acid^45^ metabolism. By contrast, higher richness attenuated *R. gnavus*’s pro-inflammatory links, suggesting that its role as a mucin glycan forager may be more pronounced in low-diversity gut environments, highlighting context-dependent and strain-dependent effects that reflect substantial intra-species genomic heterogeneity^45^.

We also confirmed that Christensenellaceae are enriched and co-occur with methanogens, and we demonstrated that this microbial constellation was enriched in LmetBMI and more strongly associated with metabolic health than with body mass per se^46^, likely through lipid-mediated effects. Similarly, several uncharacterized members of the Oscillospiraceae family were associated with favorable metabolic profiles and reduced inflammation. These associations appear to be mediated via metabolites such as N-acetylglycine, which is linked to improved adipose tissue immune tone in vivo^47^, and microbial lipids involved in intestinal cholesterol metabolism^48^.

Disentangling the effects of quantifiable obesity metrics and adjusting for bacterial gene richness revealed that metBMI residuals were primarily associated with aerotolerant, facultative anaerobic and species of oral origin—for example, *Streptococcus anginosus*—uniquely linked to systemic inflammation in our cohort and to subclinical atherosclerosis in SCAPIS^49^. Although these microbial features were also correlated with circulating levels of PPIs, their association with metBMI residuals persisted after adjusting for PPI use, suggesting that the frequently reported enrichment of oral taxa in the gut, often interpreted as a marker of preclinical disease^49^, is not solely driven by medication exposure but reflects depletion of endogenous gut commensals^50^. Notably, the enrichment of these species across the full spectrum of gene richness highlights that alterations in microbial network structure and function may be more informative than diversity metrics alone.

Taken together, our findings suggest that the gut microbiome both reflects and potentially contributes to the metabolic derangements of obesity, particularly via circulating metabolites. The metBMI signature captured a constellation of clinically relevant features, including central adiposity, insulin resistance and hypersecretion, kidney dysfunction, dietary composition and physical activity—traits not fully captured by anthropometry or standard risk assessment tools. Lack of association between PRSs and metBMI underscores environmental and lifestyle influences over genetic predisposition in shaping metabolic obesity.

Limitations of our study include its applicability to predominantly European white populations, reliance on semiquantitative metabolite data, which limits our ability to define universal ranges for the retained metabolites, and the potential exclusion of biologically relevant but non-significant findings. Although we performed mediation analyses, these do not prove biological causation. Finally, we rely on surrogate markers of insulin secretion and resistance and recognize that incorporating gold standard techniques, such as clamping for dynamic measurements, might provide more insights into metabolic obesity.

In summary, a defined, microbiome-linked metabolite panel captures the metabolic injury associated with obesity, stratifies clinical risk and predicts surgical outcomes more effectively than BMI. This signature proves robust and replicable across omics layers and cohorts, reflecting bidirectional interplay between host metabolism and the gut microbiome. Recent metabolome studies underscore the value of integrated multi-omics approaches in predicting obesity-related disease risk^8,9^, and our findings support the notion that metBMI is a more sensitive indicator of individual disease burden, particularly among individuals who fall below conventional screening thresholds.

From a translational perspective, using large-scale metabolite panels to derive obesogenic signatures is impractical in clinical settings. Our results suggest that the metBMI signature is tightly linked to insulin resistance and hypersecretion and shaped by VAT distribution and cellular characteristics. As definitions of obesity evolve, especially in light of the recent consensus to include measures of adiposity in diagnostic criteria^2^, multi-omics tools such as metBMI can provide surrogate markers and mechanistic insights into underdefined disease pathways. Among promising clinically relevant markers are dynamic insulin resistance and secretion indices, which are poorly captured by genetics alone due to their complex regulation but are essential for precision prevention and therapy. Our results lay the groundwork for experimental validation and future clinical application of this biological framework.

## Methods

### Description of study cohorts

#### IGT-microbiota cohort

We used participants from the Impaired Glucose Tolerance and Microbiota Study (IGT-microbiota), a prospective, non-interventional community-based cohort that ran between 2014 and 2018. Of 26,009 invited adults (50–65 years) without known T2D from the greater Gothenburg area, 5,152 underwent oral glucose tolerance test (oGTT), and 1,868 provided stool samples. Standardized phenotyping included anthropometrics; computed tomography-based body composition; venous blood for metabolomics, proteomics and clinical chemistry; health, lifestyle and dietary questionnaires; as well as fecal sampling, as previously described^16,51^.

Dietary intake was assessed with the MiniMealQ^52^ food frequency questionnaire (2-month reference period) to derive micronutrients/macronutrients, food items and anti-inflammatory/pro-inflammatory diet indices (AIDI and Pro-Inflammatory Diet Index (PIDI), respectively)^53^. Additional diet-related factors, including major food items and physical activity variables (total volume and total intensity)^54^, were derived from principal component analysis (PCA). Physical activity was measured using a hip-worn accelerometer (ActiGraph models GT3X+, wGT3X+ and wGT3X-BT) over 10 days and categorized as sedentary (sed), light (lpa), moderate (mpa), moderate-to-vigorous (mvpa) and vigorous (vpa)^55^, and the average time per day in that state was calculated after processing in ActiLife software.

Body composition (subcutaneous, (intra)abdominal, intermuscular and intrahepatic fat depots) was quantified from dual-source computed tomography (Siemens Medical Solutions, Somatom Definition Flash; dual-energy for the liver) as previously described^51^.

We included participants with complete clinical, metabolome and microbiome data and without known or presumed cardiovascular disease (according to history, medication or electrocardiogram), resulting in a total of 1,408 individuals (794 females and 614 males; 50–65 years of age; BMI 18.3–46.3 kg m^−^^2^, mean = 27.1 kg m^−^^2^; Extended Data Fig. 1). Multi-omics encompassed clinical laboratory tests, 1,190 metabolites, 1,462 proteins, whole fecal metagenome sequencing (over 15 million bacterial genes) and genotyping for PRSs related to body composition, BMI and lipid metabolism. Cardiovascular risk was estimated using the Framingham risk score^56^, insulin resistance by the TyG index^57^ and HOMA-IR^58^ and β cell function by HOMA-B^59^.

#### SCAPIS cohort

The validation cohort was derived from SCAPIS^51^, a prospective population-based cohort of 30,154 adults aged 50–65 years living in six municipalities between 2014 and 2018. Visits included anthropometrics, dietary questionnaires, blood draw, blood pressure measurement, fecal sampling and health/lifestyle questionnaires aligned with IGT standard operating procedures.

For validation, we analyzed data from 466 individuals with available BMI and complete metabolomics used in the metBMI model (Supplementary Table 7).

Both studies adhered to the Declaration of Helsinki with approvals from the Swedish Ethics Review Authority/regional ethics review board in Gothenburg (IGT: Swedish institutional review board study number Dnr 560-13; SCAPIS: Etikprövningsmyndigheten Dnr 2010-228-31M and Dnr 2018-315). All participants provided written informed consent, and no compensation was provided.

#### Bariatric surgery cohort

From a published cohort^20^, 189 individuals underwent metabolic surgery. Baseline data were collected 2 months prior to surgery. Exclusions were inflammatory disorders, chronic kidney disease, coronary artery disease, pregnancy or breastfeeding. A subset of 75 participants had metabolon profiling available, enabling pre-surgery metabolome-based predictions associated with 12-month outcomes. Study protocols were approved by the University of Leipzig ethics committee (applications 017-12-23012012 and 047-13-28012013), with all participants providing written informed consent.

### Data generation and preprocessing

#### Plasma metabolome

Plasma samples were randomized and profiled by Metabolon (high-performance liquid chromatography–mass spectrometry (HPLC–MS)). Processing and quality control followed established procedures with peaks identified/quantified using internal standards and software, as previously described^32^. Samples were run in 144-sample batches, and peak areas were divided by the batch’s median peak area. Metabolites were annotated against Metabolon’s library. Consistently detected but not annotated metabolites are denoted by ‘X’ followed by a unique identifier. After log transformation, batch normalization and block correction, 1,190 metabolites were retained for analysis (two metabolites missing in the entire IGT cohort, and 156 missing for 61% of the cohort). In SCAPIS, only metabolites from the main model for metBMI prediction were included, and none was missing in the validation sample.

#### Plasma proteome

Proteins were quantified with Olink PEA (1,462 proteins in four separate 384-plex panels related to inflammation, cardiometabolic disease and neurological and oncological disorders as described elsewhere)^60^. Samples were randomized. Buffer-only negatives were used to determine background and detection limits. Normalized protein expression (NPX, log_2_) was generated after quality control and normalization to standards and inter-plate plasma sample controls.

#### Genomics

Whole blood DNA was genotyped on an Illumina GSA-MDv3 array. Genotype clusters from the first batch were applied across batches for consistency (GenomeStudio 2.0.3). Quality control included checks for sex discordance, missing data, heterozygosity and batch effects. Call rate filters were ≥90% (markers/individuals), followed by a more stringent 98% call rate requirement. Hardy–Weinberg equilibrium test was performed on samples of Swedish origin at 1 × 10^−8^, and a minor allele frequency (MAF) cutoff of >0.1% was implemented. Pre-imputation harmonization was conducted using Will Rayner’s preparation script (HRC-1000Gcheck-bim-v4.3.0, https://www.chg.ox.ac.uk/~wrayner/tools/) to align strand/alleles/positions as well as frequency differences. Palindromic single-nucleotide polymorphisms (SNPs) with MAF > 0.4 were removed to mitigate the risk of allele switching, and SNPs with allele mismatches or >0.2 frequency difference between the data and the reference panel were removed. Imputation to HRC r1.1 reference panel (Sanger imputation service; EAGLE2 + PBWT) retained variants with ≥0.7 and MAF ≥ 0.01. PRSs were built using publicly available genome-wide association study (GWAS) summary statistics on the phenotypes of interest^29,30^.

#### Fecal microbiome

Participants collected chemically preserved stool samples at home using pre-packed collection kits. Samples were kept at room temperature for ≤36 hours and then stored at −80 °C at the research facility. DNA extraction and quality control followed previously described established protocols^16^. Library preparation and sequencing were performed using Illumina chemistry on HiSeq 4000 instrumentation (150-bp paired-end reads; GATC Biotech)^16^.

Reads with a Phred score less than 20 and human-mapped reads (GRCh37) were removed, yielding, on average, 26.5 million high-quality paired-end reads (range, 5.3–69 million per sample). A 15,186,403 non-redundant microbial gene catalog was assembled as previously described^16^, to which, in mean, 75.1% of reads could be mapped back (MEDUSA pipeline^61^). Gene abundance profiles across samples were rarefied to 22 million reads per sample, and mean gene abundances were obtained over 50 repeated rarefactions. Gene richness equaled the number of genes detected in the rarefied set. Taxonomic profiles were generated by mapping against the Unified Human Gastrointestinal Genome (UHGG) version 2.0 (ref. ^62^) catalog with Kraken2^63^ version 2.1.2 at the species level, and abundance profiles were estimated using Bracken^64^ 2.6.2.

BLASTX^65^ was used to derive functional annotations of the newly assembled genes against the KEGG database^66^, and the previously described customized GMM set was expanded by six trimethylamine (TMA) and 20 phenylpropanoid metabolism modules^17,67^. Omixer-RPM^68^ version 1.1 was used for GMM abundance computation with module presence requiring ≥60%, as detailed elsewhere^17^.

### Statistical analyses

#### Analyses of variance explained

Variances explained for each covariate were estimated using both ridge and LASSO regression with nested 10-fold cross-validation (glmnet version 4.1.6). The final results are based solely on ridge regression, as both methods yielded similar performance. Still, BMI prediction with ridge regression yielded a slightly improved prediction (Methods: ‘Ridge and LASSO regression on BMI’). Ridge regression models were conducted in the 1,408 study participants, excluding those with missing data, using microbial species abundances (MAGs, center log ratio (CLR) transformed), scaled GMMs, KEGG modules, metabolomics, proteomeomics, diet and metadata. Feature spaces focused on BMI and adiposity measures (waist circumference, WHR, areas and attenuations for abdominal VAT attenuation and SAT), microbiome richness and other omics space variables.

In each nested iteration, nine folds were used to train a ridge model with an inner 10-fold grid search to identify the optimal lambda value. The test fold (held-out fold) was then used to calculate the out-of-bag prediction, *R*^2^ and the test error. When predicting a variable, the entire feature space containing that variable was excluded (for example, no metabolome data were used to predict single circulating metabolites).

#### Ridge and LASSO regression on BMI

BMI was modeled with ridge and LASSO regression. Only metabolites significantly associated with BMI (Spearman’s *ρ* > 0.1) were included in the model. To balance the sex and BMI groups, equal numbers were sampled from the World Health Organization (WHO) BMI categories (BMI 18.5–24.9 kg m^−2^, 25–29.9 kg m^−2^ and ≥30 kg m^−2^), limited by the smallest stratum (129 men with a BMI of 18.5–24.9 kg m^−2^), yielding 774 individuals. These were split randomly into a 75% training set and a 25% test set. Remaining participants not within the BMI bins were allocated to the ‘non-test’ set, which, together with the test set, constituted the ‘extended test set’. After *λ*-parameter optimization, ridge regression was performed using cv.glmnet with 10-fold cross-validation to minimize the mean squared error (glmnet version 4.1.6, length *λ* = 100, range 10^−3^ to 10^−5^). Hold-out *R*^2^ on the BMI-binned test set quantified performance. Ridge and LASSO achieved *R*^2^ = 0.39 and *R*^2^ = 0.35, respectively. The final ridge model was used to predict BMI (henceforth, metBMI) in the entire cohort. Residuals from a model adjusting for age, sex and BMI were extracted for further downstream analyses. Participants with residuals < −2.5 were classified as having a predicted metabolic BMI lower (LmetBMI, *n* = 147), and participants with residuals > 2.5 were classified as having a higher predicted than their ground truth BMI (HmetBMI, *n* = 154). Others were classified according to their WHO BMI categories: normal weight (*n* = 313), overweight (*n* = 488) or with obesity (*n* = 307). Residual distributions were similar across training and test sets and BMI categories. MetBMI was modestly lower at very high BMI (Extended Data Fig. 3d).

#### Logistic regression for disease prevalence

Associations between binary cardiometabolic outcomes and BMI or metBMI were assessed using logistic regression, adjusted for age and sex (binomial glm, stats version 4.1.1). For each outcome, three models were constructed: one that included BMI, one that included metBMI and a nested model that included both. Independence from conventional risk factors was tested in a second set, adjusting for WHR, HDL, LDL, triglycerides, systolic and diastolic blood pressure, glucose and statin use. The added value of metBMI beyond BMI was tested using LRTs (ANOVA function) that compared nested models with BMI-only models.

#### PCA on metabolite levels

PCA on the complete metabolomics data was performed using prcomp and visualized using fviz_eig (factoextra version 1.0.1 (ref. ^69^)). Resulting Euclidean distances were extracted and plotted using ggplot2 version 3.4.0 (ref. ^70^).

#### Correlations and regression

Partial Spearmanʼs correlations for gene richness and metabolic BMI residuals were used to derive estimates adjusted for age, sex and BMI and multiple testing (ppcor version 1.1, p.adjust in stats version 4.1.1 at 5% FDR). The categorical sex variable was converted into a dummy variable prior to analysis. Linear regression models of gene richness included diabetes status, MetS presence^71^ and BMI as independent variables in one model. MetBMI was added in a second model. *P* values were obtained using the *F-*test, and *P* < 0.05 was considered significant. Similarly, individual linear regressions of gene richness against available variables were performed iteratively, correcting for BMI, age and sex or metBMI, age and sex. Variables with near-zero variance (estimated using caret version 6.0.93 (ref. ^72^))—for example, N-acetyl sulfapyridine, rocuronium, rivaroxaban, cefazolin and X-21628—were excluded from the model. Normality was assessed with the Andersen–Darling test (nortest version 1.0.4 (ref. ^73^)), and non-normally distributed variables were log transformed.

#### RFE and bidirectional mediation analysis

To refine the variables for subsequent downstream analyses, including bidirectional mediation, we implemented RFE on the metabolome and metadata datasets. These datasets comprised variables related to diet, physical activity, clinical chemistry and anthropometry, including body composition. We applied Boruta (version 8.0.0 (ref. ^74^), 999 importance source runs). This process narrowed down the most pertinent metabolites to 66, with 10 consistently selected in iterative LASSO models across all tested models, 51 identified in the ridge regression model and five additional metabolites (Supplementary Table 15). Similarly, the clinical features tested from the metadata were reduced to 63 variables.

For mediation, we first computed Spearmanʼs correlations among (1) microbiome species overlapping across obesity traits and associated with metBMI residuals after adjusting for other obesity measures and richness (68 species) and (2) 57 GMMs associated with metBMI residuals. We then correlated these with (3) the 66 metabolites and (4) the 63 metadata variables identified through RFE. We retained only those variables from the three feature sets that exhibited significant correlations with variables from the other sets, adhering to a minimum absolute Spearmanʼs correlation threshold of 0.1 and a maximum adjusted *P* value threshold of 0.05, following the Benjamini–Hochberg correction.

As a result, 66 bacterial species, 51 GMMs, 56 metabolites and all 63 metadata variables were kept for further mediation analysis. Using these variables, three grids containing all possible variable combinations were constructed. The combinations were arranged in the following sequence: microbiome feature → metabolite → phenotype variable; microbiome feature → phenotype variable → metabolite; and phenotype variable → metabolite → microbiome feature. These sequences were used to test for direct and reverse mediation effects for microbiome features via metabolites and phenotypes, respectively, and to assess reverse causation in the third configuration.

The mediation analysis was conducted separately for each grid by fitting the model *y* = *x* + *m*, where ‘*y*’ is the outcome variable (phenotype in direct mediation and metabolite in reverse mediation), ‘*m*’ is the mediator (metabolite in direct mediation and phenotype in reverse mediation) and ‘*x*’ is the exposure variable (microbiome feature in both direct and reverse mediations). In the third mediation grid, ‘*y*’ represents the microbiome feature, and ‘*x*’ represents the phenotype variable. Unstandardized indirect effects were computed (mediation version 4.5.0 (ref. ^75^), 1,000 bootstrap). The average causal mediation effect (ACME), reflecting the isolated effect of the mediator, was determined for each direction, and its *P* values were adjusted for multiple comparisons using the Benjamini–Hochberg method.

Microbiome–phenotype linkages via metabolites were identified after excluding linkages with reverse mediation and direct phenotype–bacteria effect by FDR-ACME < 0.05, bacteria → phenotype → metabolite (*P* value-ACME.inverse > 0.05), phenotype → metabolite → bacteria (*P* value-ACME.inverse2 > 0.05) as well as phenotype → bacteria (*P* value-average direct effect (ADE) > 0.05). Microbiome–metabolome linkages via phenotypes were established based on FDR-ACME.reverse1 < 0.05, *P* value-ACME > 0.05, *P* value-ACME.reverse2 > 0.05 and *P* value-ADE.reverse2 > 0.05. Phenotype–bacteria linkages, either direct or via metabolites, were identified with *P* value-ACME and *P* value-ACME.reverse1 > 0.05 and FDR-ACME.reverse2 and/or FDR-ADE.reverse2 < 0.05 for mediated effect or combined mediated and direct effects, respectively.

### Microbiome analyses

Species-level data were filtered at 5% prevalence filter and combined into a phyloseq object (phyloseq version 1.42.0 (ref. ^76^), 2,820 unique taxa/MAGs from 1,408 samples, 22 phyla and 790 genera, non-filtered: 3,331). PCA was performed using metric multidimensional scaling (MDS) and Aitchison distances on CLR-transformed taxa counts, constructed with the vegdist function from vegan. Adonis2 was used to estimate the contribution of the metBMI group to the community variation, followed by a pairwise multilevel comparison using the wrapper pairwise.adonis with Bonferroni adjustment (Supplementary Table 19).

Differential abundance analyses were performed at the species level using ANCOM-BC version 1.4.0 (ref. ^77^) with covariates age and sex added to the formula (FDR < 0.05). We then evaluated medication confounding on the reported differentially abundant features using metadeconfoundR^18^, reporting only non-confounded (that is, no impact of the confounder) or strictly de-confounded (that is, the effect of the variable is independent of the confounder) features at an FDR of ≤0.1. Overall, the effect sizes and their direction were congruent between ANCOM-BC and metadeconfoundR, and all significant features reported in ANCOM-BC displayed a significant effect size in metadeconfoundR. MetadeconfoundR was similarly used to elucidate whether the effect of a particular variable (for example, metBMI residuals) on a specific taxon was more closely related to another obesity measure. Similarly, gene richness was included as a predictor to understand whether changes in overall gene richness underlie differentially abundant features or whether these are indeed unlinked to the general loss of richness observed in obesity and metabolic health deterioration. Low-abundant taxa were defined as less than 5% of the mean total abundance. Differential abundance of rarefied GMMs was conducted along with de-confounding directly in metadeconfoundR^18^.

Partial Spearman’s ranked-sum correlations are reported between the overlapping 46 differentially abundant taxa in all four obesity features and other covariates. Heatmaps were produced using the package ComplexHeatmap^78^ and show only taxa with at least one significant correlation in the set of metadata variables given at an FDR-adjusted significance of less than 0.1. Tiles showing FDR < 0.01 and FDR < 0.05 are depicted with ‘*’ and ‘+’, respectively.

#### Correlations and metadeconfoundR analysis for species–host associations

We computed Spearmanʼs correlations between selected microbial species and host features (metabolites, diet, physical activity and clinical metrics), retaining associations with FDR < 0.1 and Spearman’s *ρ* > 0.1. A subsequent metadeconfoundR^18^ analysis was employed to filter associations that were unconfounded by other variables, including gene richness.

#### Comparative microbiota network analysis

Signed networks were constructed using NetCoMi version 1.1.0 (ref. ^79^) using the 500 species exhibiting the highest variance in HmetBMI and LmetBMI subsets. Associations were analyzed using a two-sided Spearmanʼs correlation with a threshold of 0.3 after total sum scaling (TSS) normalization and multiplicative zero replacement. Network properties were analyzed and visualized using the netAnalyze function. A differential network was constructed using the diffnet function, with Fisher tests and local FDR adjustment.

### Reporting summary

Further information on research design is available in the Nature Portfolio Reporting Summary linked to this article.

## Online content

Any methods, additional references, Nature Portfolio reporting summaries, source data, extended data, supplementary information, acknowledgements, peer review information; details of author contributions and competing interests; and statements of data and code availability are available at 10.1038/s41591-025-04009-7.

## Supplementary information

Reporting Summary

Supplementary Table 1–19 Supplementary Tables 1–19.

## Data Availability

The IGT-microbiota and SCAPIS deidentified datasets used in this study are accessible to qualified researchers via a data use agreement for research purposes after consideration from the data accession committee. For data access inquiries, please contact Fredrik Bäckhed; responses will be provided within seven business days. The raw whole metagenome shotgun (WMGS) data are available upon reasonable request. Whole metagenomic data are deposited at the European Nucleotide Archive under accession numbers PRJEB100670 and ERP174669.

## Code availability

No specialized in-house code was used for this study. All software used for the data analyses in this study is publicly available and cited in Methods.
