# Supplementary material for: Multi-omic definition of metabolic obesity through adipose tissue–microbiome interactions
Source: Nat Med. 2026 Jan 2;32(1):113–25. doi: 10.1038/s41591-025-04009-7 (PMC12823436; doi:10.1038/s41591-025-04009-7)
Supplement: Supplementary file 1 — Reporting Summary [file 41591_2025_4009_MOESM1_ESM.pdf]

nature portfolio

Corresponding author(s): Rima Chakaroun, Fredrik Bäckhed

Last updated by author(s): Aug 15, 2025



# Reporting Summary

Nature Portfolio wishes to improve the reproducibility of the work that we publish. This form provides structure for consistency and transparency in reporting. For further information on Nature Portfolio policies, see our Editorial Policies and the Editorial Policy Checklist.

## Statistics

For all statistical analyses, confirm that the following items are present in the figure legend, table legend, main text, or Methods section.

| n/a | Confirmed | |
|---|---|---|
| ☐ | ☒ | The exact sample size (*n*) for each experimental group/condition, given as a discrete number and unit of measurement |
| ☐ | ☒ | A statement on whether measurements were taken from distinct samples or whether the same sample was measured repeatedly |
| ☐ | ☒ | The statistical test(s) used AND whether they are one- or two-sided<br>*Only common tests should be described solely by name; describe more complex techniques in the Methods section.* |
| ☐ | ☒ | A description of all covariates tested |
| ☐ | ☒ | A description of any assumptions or corrections, such as tests of normality and adjustment for multiple comparisons |
| ☐ | ☒ | A full description of the statistical parameters including central tendency (e.g. means) or other basic estimates (e.g. regression coefficient) AND variation (e.g. standard deviation) or associated estimates of uncertainty (e.g. confidence intervals) |
| ☐ | ☒ | For null hypothesis testing, the test statistic (e.g. *F*, *t*, *r*) with confidence intervals, effect sizes, degrees of freedom and *P* value noted<br>*Give P values as exact values whenever suitable.* |
| ☒ | ☐ | For Bayesian analysis, information on the choice of priors and Markov chain Monte Carlo settings |
| ☒ | ☐ | For hierarchical and complex designs, identification of the appropriate level for tests and full reporting of outcomes |
| ☐ | ☒ | Estimates of effect sizes (e.g. Cohen's *d*, Pearson's *r*), indicating how they were calculated |

*Our web collection on statistics for biologists contains articles on many of the points above.*

## Software and code

Policy information about availability of computer code

| | |
|---|---|
| Data collection | FINDRISC was used for calculating the diabetes risk and MiniMealQ for collecting the dietary data. Cardiovascular risk was estimated using the Framingham risk score and physical activity was measured using a hip-worn accelerometer (ActiGraph model GT3X+, wGT3X+, and wGT3X-BT, ActiGraph LCC, Pensacola, FL, USA). |
| Data analysis | Open-source and standard packages include: Kraken2 (v2.1.2) and Bracken (v2.6.2), MEDUSA for microbial gene abundance estimation, BLASTX, Omixer-RPM (v1.1), glmnet (v4.1.6), ppcor (v1.1),nortest (v1.0.4), caret (v6.0.93), Boruta (v8.0.0), mediation (v4.5.0), phyloseq (v1.42.0) and vegan, ANCOM-BC (v1.4.0), metadeconfoundR, ComplexHeatmap, NetCoMi (v1.1.0), and ggplot2 (v3.4.0) and factoextra (v1.0.1). All statistical analyses and visualizations were conducted in R (version 4.1.1) |

For manuscripts utilizing custom algorithms or software that are central to the research but not yet described in published literature, software must be made available to editors and reviewers. We strongly encourage code deposition in a community repository (e.g. GitHub). See the Nature Portfolio guidelines for submitting code & software for further information.

## Data

Policy information about availability of data

All manuscripts must include a data availability statement. This statement should provide the following information, where applicable:

- Accession codes, unique identifiers, or web links for publicly available datasets
- A description of any restrictions on data availability
- For clinical datasets or third party data, please ensure that the statement adheres to our policy

The IGT-microbiota and SCAPIS de-identified datasets used in this study are accessible to qualified researchers via a Data Use Agreement for research purposes. For data access inquiries, please contact Prof. Fredrik Bäckhed; responses will be provided within seven business days. The raw WMGS data are available upon request. whole metagenomic data are deposited at ENA under accession numbers PRJEB91733 and ERP174669.

## Research involving human participants, their data, or biological material

Policy information about studies with human participants or human data. See also policy information about sex, gender (identity/presentation), and sexual orientation and race, ethnicity and racism.

| | |
|---|---|
| Reporting on sex and gender | The study includes both sexes. Sex (based on self-reporting) was used throughout the study and included as covariate for modelling. sex-specific post hoc analyses were performed |
| Reporting on race, ethnicity, or other socially relevant groupings | the term European is used to describe participants in this study. |
| Population characteristics | The study has included the population-based cohorts (IGT and SCAPIS) from Gothenburg, Sweden and a bariatric surgery cohort from Germany. Population characteristics are described in the manuscript in Suppelementary Tables. |
| Recruitment | As a population level study, all participants were randomly invited by letter and thus without any specific selection that might impact the results. All invited participants were screened with questionnaire and oral glucose tolerance test. participants undergoing bariatric surgery had to be elligible for surgery according to clinical criteria. |
| Ethics oversight | all studies adhered to the ethical guidelines of the Declaration of Helsinki and were approved by the regional ethics review board in Gothenburg (IGT: the Swedish Ethics Review Authority, institutional review board study number Dnr560-13) and Swedish Ethics Review Authority (SCAPIS: Etikprövningsmyndigheten Dnr 2010-228-31M, Dnr 2018-315). All participants provided written informed consent, and no compensation was provided for their participation. For bariatric surgery The study protocols were approved by the University of Leipzig's ethics committee (applications 017-12-23012012 and 047-13-28012013), with all participants providing written informed consent. |

Note that full information on the approval of the study protocol must also be provided in the manuscript.

# Field-specific reporting

Please select the one below that is the best fit for your research. If you are not sure, read the appropriate sections before making your selection.

☒ Life sciences ☐ Behavioural & social sciences ☐ Ecological, evolutionary & environmental sciences

For a reference copy of the document with all sections, see nature.com/documents/nr-reporting-summary-flat.pdf

# Life sciences study design

All studies must disclose on these points even when the disclosure is negative.

| | |
|---|---|
| Sample size | The complete IGT microbiota cohort included 1833 individuals. Only participants with CT-scans and complete multi-omics were included, leaving 1408 participants. No a priori Power calculation was conducted. in our previous publication, a subset of this cohort was deemed to have sufficient power for modest effect sizes (DOI: 10.1016/j.cmet.2020.06.011). |
| Data exclusions | participants without CT-scans, Microbiome and multi-omics data were excluded from IGT-microbiota and SCAPIS. |
| Replication | The SCAPIS cohort was used for replication. |
| Randomization | As a cohort-based observational study, the sample collection and measurements were performed in a random order with no further randomization. |
| Blinding | As a cohort-based observational study, no blinding was performed. |

# Reporting for specific materials, systems and methods

We require information from authors about some types of materials, experimental systems and methods used in many studies. Here, indicate whether each material, system or method listed is relevant to your study. If you are not sure if a list item applies to your research, read the appropriate section before selecting a response.

## Materials & experimental systems

| n/a | Involved in the study |
|---|---|
| ☒ | ☐ Antibodies |
| ☒ | ☐ Eukaryotic cell lines |
| ☒ | ☐ Palaeontology and archaeology |
| ☒ | ☐ Animals and other organisms |
| ☒ | ☐ Clinical data |
| ☒ | ☐ Dual use research of concern |
| ☒ | ☐ Plants |

## Methods

| n/a | Involved in the study |
|---|---|
| ☒ | ☐ ChIP-seq |
| ☒ | ☐ Flow cytometry |
| ☒ | ☐ MRI-based neuroimaging |

## Plants

Seed stocks: n/a

Novel plant genotypes: n/a

Authentication: n/a
